# Discovery and preclinical development of a SdAb-based CAR-T technology for targeting CD33 in AML

**DOI:** 10.1016/j.omton.2025.200949

**Published:** 2025-02-11

**Authors:** Franco Bernasconi-Bisio, Eva Molina, Vianca Ibarra, Inés Ibáñez-Sala, Federica Rochira, Patricia Jauregui, Saray Rodríguez-Diaz, Rebeca Martínez-Turrillas, Iñigo Azagra-Barber, Nuria Gómez-Cebrián, Juan José Lasarte, Leonor Puchades-Carrasco, Lucía Vanrell, Juan Roberto Rodríguez-Madoz, Felipe Prósper, Antonio Pineda-Lucena

**Affiliations:** 1 Therapeutic Innovation Program, Cima Universidad de Navarra, 31008 Pamplona, Spain; 2 Hemato-Oncology Program, Cima Universidad de Navarra, IdiSNA, 31008 Pamplona, Spain; 3 Immunology and Immunotherapy Program, Cima Universidad de Navarra, IdiSNA, 31008 Pamplona, Spain; 4 Nanogrow Biotech, Montevideo 11500, Uruguay; 5 Hematology and Cell Therapy Department, Clínica Universidad de Navarra, IdiSNA, 31008 Pamplona, Spain; 6 Cancer Center Clínica Universidad de Navarra (CCUN), 31008 Pamplona, Spain; 7 Centro de Investigación Biomédica en Red de Cáncer (CIBERONC), 31008 Pamplona, Spain; 8 Drug Discovery Unit, Instituto de Investigación Sanitaria La Fe (IIS La Fe), 46026 Valencia, Spain

**Keywords:** MT: Regular Issue, single-domain antibodies, SdAbs, nanobodies, VHH libraries, CAR-T cell therapy, acute myeloid leukemia, AML, CD33

## Abstract

Chimeric antigen receptor T cell (CAR-T) therapies have revolutionized cancer immunotherapy. Traditional single-chain variable fragments (ScFvs) used as CAR recognition moieties face challenges such as high tonic signaling, compromised binding epitopes, and suboptimal affinity. Single-domain antibodies (SdAbs) offer an attractive alternative due to their smaller size, stability, and reduced immunogenicity. In this work, we developed an SdAb-CAR-T cell discovery platform integrating generation, characterization, and selection of SdAbs based on various properties. This approach was demonstrated by developing CAR-T cells with SdAbs against CD33, a target for acute myeloid leukemia (AML). We identified diverse SdAbs against CD33, with affinities ranging from 3.9–115 nM, and characterized their binding kinetics and epitope recognition. Using SdAb-based second-generation CARs, we assessed tonic signaling, T cell phenotypes, cytotoxicity and cytokine release *in vitro*, resulting in reduced tonic signaling and increased cytokine production. *In vivo*, SdAb-based CAR-T cells exhibited enhanced efficacy at lower doses, in a xenograft AML mouse model, demonstrating advantages over ScFv-based CD33 CAR-T cells.

## Graphical abstract

**Figure:**
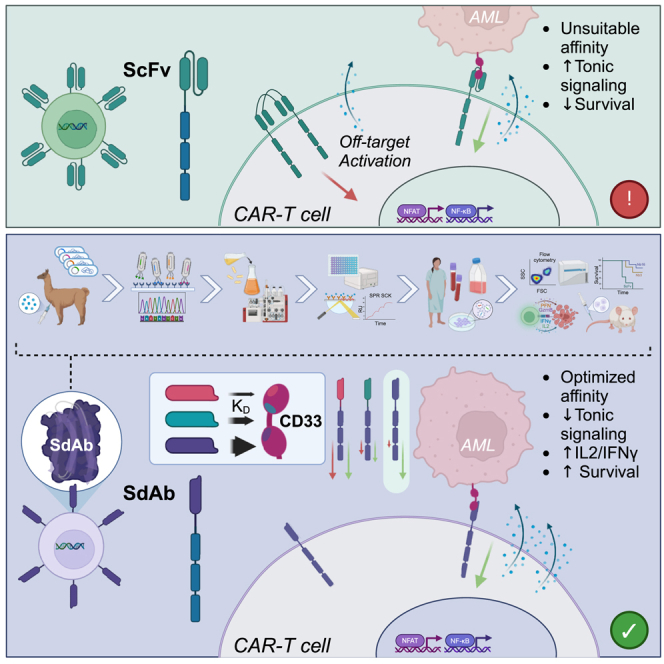

## Teaser

Bernasconi-Bisio, Pineda-Lucena and colleagues established an integrative SdAb platform for CAR-T cell development addressing limitations of a conventional ScFv-based CAR against CD33 for AML targeting. SdAb-based CARs overcome challenges like tonic signaling, suboptimal affinity, cytokine production and limited survival of AML xenografts potentially enhancing the efficacy of CAR-T cell therapy.

## Introduction

Adoptive transfer of engineered chimeric antigen receptor (CAR)-modified T cells^1^ has revolutionized the treatment of hematological malignancies, particularly B cell malignancies, such as lymphoma, acute lymphoblastic leukemia (ALL), and multiple myeloma (MM).^2^^,^^3^^,^^4^ Currently, most approved CAR-T cell products use single-chain variable fragments (ScFv) as the antigen recognizing moiety.^5^ Despite their excellent binding affinity and specificity, ScFvs have several limitations, including the possibility of self-aggregation leading to CAR clustering, tonic signaling, and eventually T cell exhaustion.^6^^,^^7^ Additionally, the use of ScFvs derived from mice or the inclusion of artificial linkers can induce immunogenicity, thereby reducing efficacy.

An alternative to ScFvs fragments is the use of camelid-derived single-domain antibodies (SdAbs), also known as Nanobodies (Ablynx). SdAbs are small antibody fragments derived from a special type of antibodies present in camelids, known as “heavy chain only antibodies” (HcAbs). The variable domain of HcAbs, known as VHH, harbors the entire paratope.^8^^,^^9^ SdAbs have proven to be promising targeting moieties for CAR-T cells due to their small size, high stability, and reduced receptor aggregation or V_H_-V_L_ mispairing, which mitigates excessive tonic signaling and T cell exhaustion.^10^^,^^11^ SdAbs can be obtained from VHH libraries, such as those derived from immunized camelids, and are easy to clone and adaptable to many display platforms, like phage display, allowing the isolation of specific SdAb clones against a target of interest.^12^ By harnessing SdAbs with diverse affinities and binding epitopes, the specificity and efficacy of CAR-T therapy can be enhanced while minimizing off-target effects, T cell exhaustion, poor T cell persistence, and antigen escape.

In the current study, we describe the isolation and characterization of a diverse array of llama SdAbs against the myeloid marker CD33 (Siglec-3)^13^ and the development of SdAb-based CAR-T cells as a potential treatment for acute myeloid leukemia (AML). By immunizing an adult *Lama glama* with the extracellular domain (ECD) of human CD33, we were able to isolate a large number of unique SdAb binders and assess binding affinity and kinetics, as well as epitope competition, to select promising candidates for CAR design. Through a series of selective experiments, we evaluated CAR activation profiles, as well as *in vitro* and *in vivo* functionality in a xenograft AML model, comparing these with benchmark CD33-CAR-T cells based on ScFv. Overall, the results support the evaluation of the clinical development of CD33-targeted SdAb-based CAR-T therapeutics for patients with AML.

## Results

### Identification, selection, and biophysical characterization of CD33-specific SdAbs

**Figure 1:**
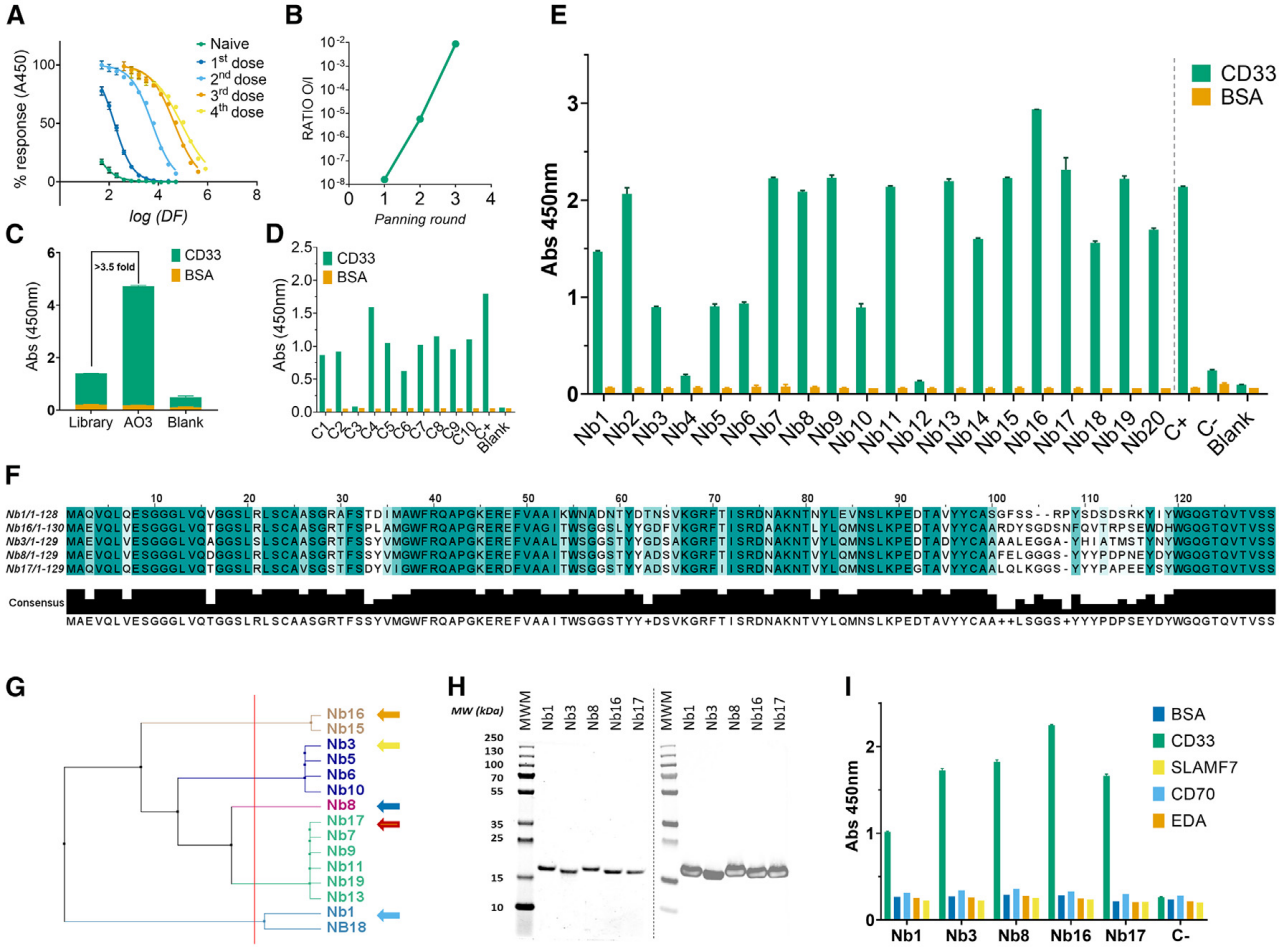
Identification of CD33-specific SdAbs (A) CD33 indirect ELISA curves of llama plasma at different stages during immunization (percentage of signal vs. plasma dilution factor [DF]). (B) Output/input (O/I) phage ratio for each panning round. (C) Phage ELISA of the original library and the final output AO3. (D) Preliminary ELISA screening of isolated *E. coli* clones infected with bacteriophages. As positive control, one positive clone from a previous round output (C+) was offered. (E) Final ELISA screening of isolated *E. coli* clones expressing the SdAbs from the expression vector pETMod. The positive control (C+) was C4 from the preliminary screening, and the negative control was an irrelevant SdAb-expressing clone culture supernatant from a panning round against another target. (F) Sequence alignment of the five candidate SdAbs selected for further characterization. (G) Phylogenetic tree of the identified SdAb sequences against CD33, clustered into five families based on half distance. (H) Coomassie blue-stained SDS-PAGE and anti-HA Western blot of the IMAC and IEC purified SdAbs. (I) Cross reactivity test (ELISA) of the five candidate SdAbs against antigens from the same llama library.

A phage display SdAb library with a diversity of 1.75 × 10^10^ colony-forming units (CFU), and phage titers exceeding 10^1^³ CFU, was generated from an immunized llama with a specific humoral response against CD33 (half maximal effective concentration [EC_50_] of 1 of 108,591) (Figure 1A). After three rounds of panning, phage enrichment was achieved, as indicated by an increase in the output/input (O/I) ratio (Figure 1B), a heightened signal in the M13 polyclonal phage ELISA (Figure 1C), and a substantial representation of positive clones in preliminary monoclonal screening (Figure 1D). The amplified phagemids from the final panning round were subcloned into an expression vector and transformed into *E. coli* BL21 for clone isolation. Of 20 isolated colonies, 18 clones (90%) were positive against CD33 and were subsequently sequenced (Figure 1E). Phylogenetic analyses of identified sequences, based on the mean distance between sequences (Figure 1F), revealed five different clustered families (Figure 1G). Given that sequence dissimilarities often reflect diverse binding epitopes and affinities, thereby increasing the likelihood of obtaining SdAbs with varying properties, a representative SdAb from each family (Nb1, Nb3, Nb8, Nb16, and Nb17) were selected, produced, and purified, showing excellent purity and specific activity (Figures 1H and 1I).

**Figure 2:**
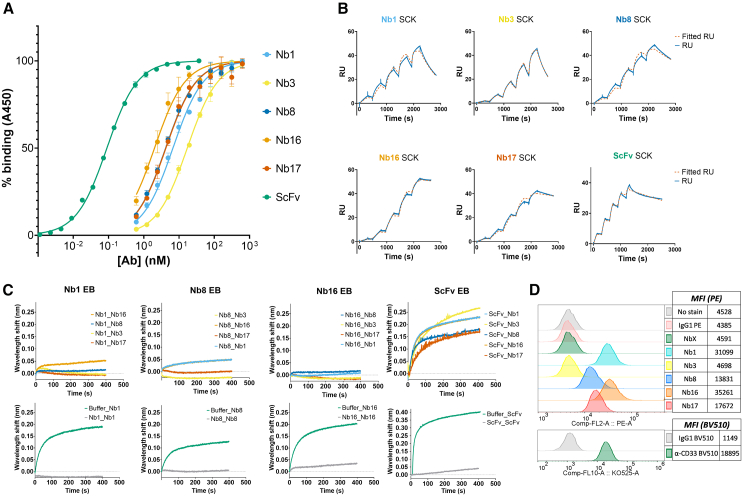
Characterization of SdAbs (A) ELISA sigmoidal curves for each candidate SdAb and the “My96” ScFv as relative A450 vs. log of SdAb concentration (nM). (B) SPR sensorgrams for each SdAb and the reference ScFv from SCK runs, along with their respective fitting curves (χ^2^ < 10% Rmax, tc > 100∗Kon, and U < 15) (C) BLI epitope binding curves of the secondary binding of each SdAb, the primary SdAb or ScFv to bind is specified in each graph title. (D) Histograms of anti-HA PE stained MOLM13 CD33+ cells pre-incubated with 1 μg of each SdAb for 1 h, alongside control staining with antiCD33 BV510 (WM53).

To characterize binding affinity and kinetics of each SdAb, ELISA and surface plasmon resonance (SPR) were performed. The SdAb candidates and the reference ScFv exhibited varying EC_50_ values in ELISA (Figure 2A), corresponding to their relative affinities (Table 1). Binding kinetics, determined via SPR (Figure 2B), allowed the determination of k_on_, k_off_, and K_D_ values (Table 1). The affinities assessed by SPR correlated with the EC_50_ values observed in ELISA. Among the SdAb candidates, Nb16 displayed the highest affinity (K_D_ = 4.18 nM), while Nb3 exhibited the lowest affinity (K_D_ = 115 nM). The reference ScFv showed a K_D_ of 0.35 nM. To explore whether selected SdAbs candidates might compete for binding epitopes on CD33, biolayer interferometry (BLI) epitope binning was performed using the ScFv and the SdAbs exhibiting the lowest dissociation rates in BLI (Nb1, Nb8, and Nb16) as first binders. As shown in Figure 2C, varying degrees of partial or complete competition among SdAbs for CD33 binding were observed. Partial competition was noted for Nb16 with Nb8 and Nb1, and between Nb8 and Nb1. All other SdAb combinations exhibited nearly complete competition, suggesting epitope overlapping. These observations were further supported by *in silico* modeling, where SdAb structures were docked to the CD33 ectodomain PDB structure (Figure S2). In contrast, the ScFv did not compete with any of the SdAbs for CD33 binding, indicating that its epitope is different from that of the SdAbs. To determine if SdAbs could bind to CD33 naturally expressed on the surface of AML cells, a flow cytometry binding assay was performed. We observed that selected SdAbs bind CD33, exhibiting a CD33-dependent pattern similar to that of the anti-CD33 mouse monoclonal antibody (clone WM53) (Figure 2D). Nb3 showed limited binding, likely due to its low affinity and rapid dissociation from the target (k_off_ = 1.70 E−03 s^−1^), which may not be optimal for flow cytometry staining conditions.

**Table 1:** CD33 binding affinity and kinetic constants for the five SdAb candidates and the reference ScFv

Antibody	EC_50_ (M)	K_D_ (M)	k_on_ (M^−1^s^−1^)	k_off_ (s^−1^)
Nb1	6.40E−09	2.99E−08	3.68E+04	1.10E−03
Nb3	1.67E−08	1.15E−07	1.47E+04	1.70E−03
Nb8	3.99E−09	1.44E−08	2.48E+04	3.56E−04
Nb16	1.91E−09	4.18E−09	1.11E+04	4.64E−05
Nb17	4.05E−09	4.99E−09	1.65E+04	8.24E−05
ScFv	8.99E−11	3.52E−10	5.12E+05	1.81E−04

### Functional characterization of SdAb-based CAR-T cells against CD33

**Figure 3:**
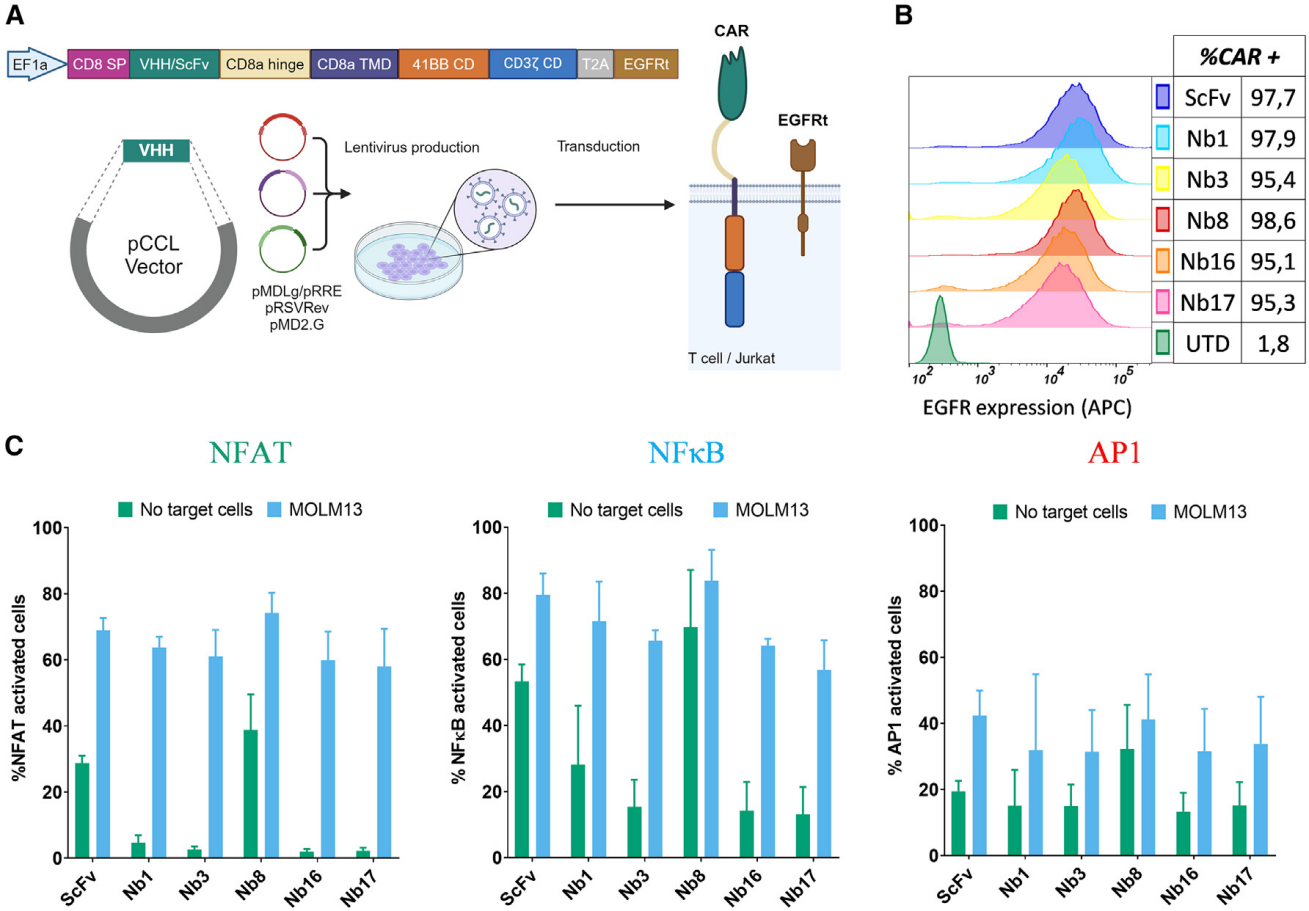
SdAb-based CAR constructs activation profiles (A) Schematic representation of the second-generation SdAb-based CAR construct design, the generation of lentiviral vectors, and the final CAR expressed on T cells or Jurkat cells, created with BioRender.com. (B) Histograms of EGFR staining representing CAR expression in Jurkat-TPR cells. Histograms represent MFI. (C) Activation profile of the SdAb/ScFv based CAR-expressing Jurkat-TPR cells expressed as percentage of positive events for each activation pathway (mean and SD, *n* = 3). From left to right: NFAT (GFP-reported), NF-κB (BFP-reported) and AP1 (mCherry-reported) mediated activation of untreated cells (green bars) and co-cultured with MOLM13 in a 1:1 ratio (light blue bars).

First, we assessed the potential of newly identified SdAbs as recognition moieties in second-generation CAR, assembled within a third-generation lentiviral vector (Figure 3A) using the Jurkat reporter (TPR) cell line. Jurkat cells were transduced with all CAR constructs, including a previously described ScFv CD33-CAR (Figure 3B). Generally, SdAb-based CARs exhibited low activation of NFAT, NF-κB, and AP1 pathways in the absence of CAR stimulation (tonic signal), except for Nb8 CAR, which displayed similar or even higher levels than the control ScFv-CAR. SdAb-CAR constructs responded to CD33+ MOLM13 cells with activation of the three pathways, with NFAT and NF-κB being the most strongly activated (Figures 3C and S3). Notably, except for Nb8-CAR, all Nb-based CARs demonstrated the highest activation ratios for both the NFAT and NF-κB pathways in the presence of CD33+ MOLM13 cells compared with activation without target cells. We did not find a clear correlation between SdAb affinity and the activation profile, as Nb16 and Nb3, with contrasting affinities (highest and lowest, respectively) exhibited similar CAR activation profiles compared with that of Nb8, which has an intermediate K_D_ value. Based on these results and the need for diverse affinities, we selected Nb16-, Nb3-, and Nb1-based CAR constructs, which displayed low tonic signaling and represented high, low, and intermediate binding affinities, respectively, for further evaluation in human T cells.

**Figure 4:**
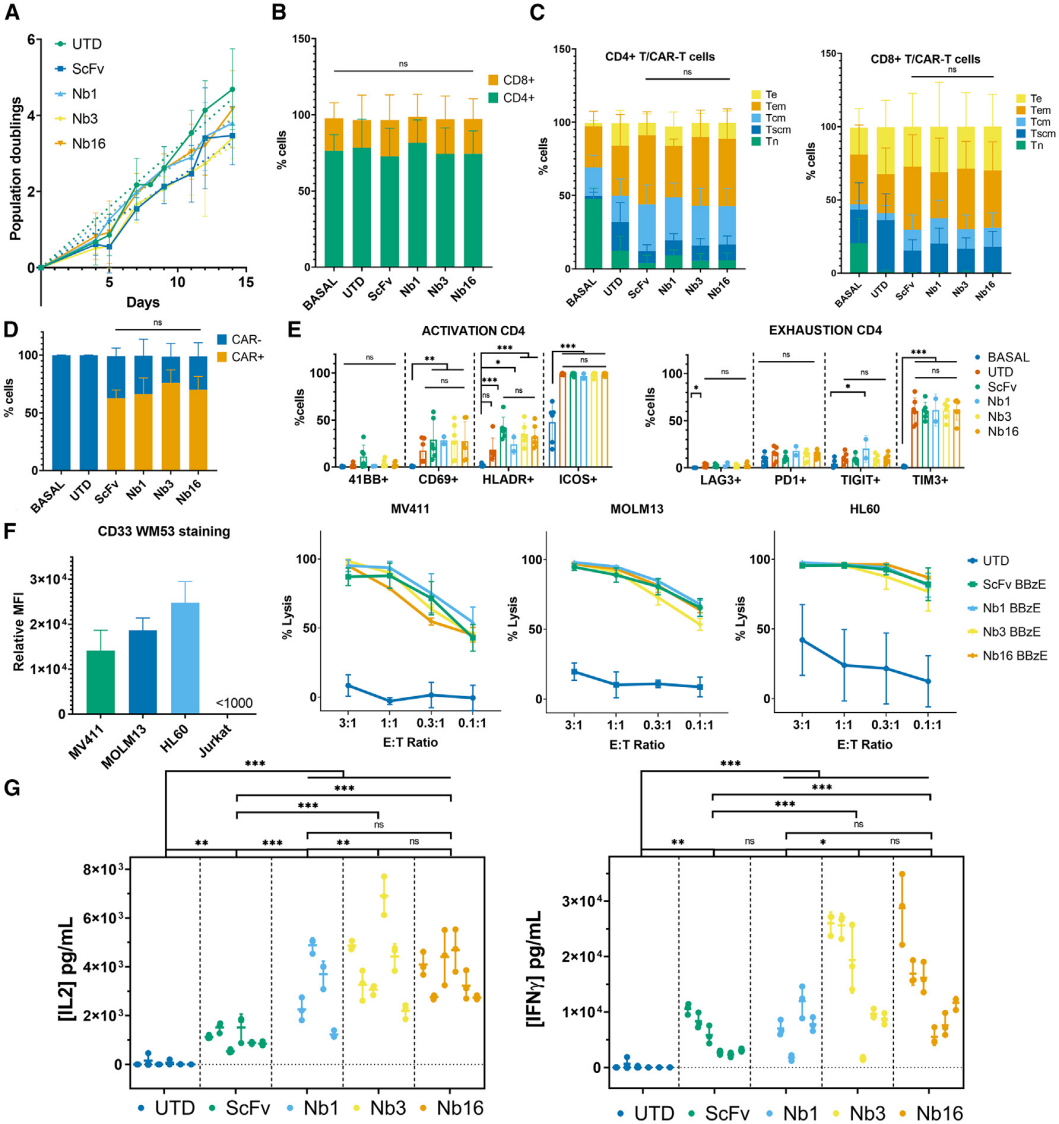
Functional characterization of SdAb-CAR-T cells (A) Population doublings during 14 days of CAR-T cell expansion. (B–D) FACS characterization of untransduced T cells (UTD) and CAR-T cells at baseline and after 12–14 days. Repeated measures ANOVA with Tukey’s multiple comparisons (FDR correction) was performed for all compositional analyses. (B) T cell populations (CD8/CD4) and (C) subpopulations (Te: effector, Tem: effector memory, Tcm: central memory, Tscm: stem cell memory, Tn: naive) of CD4 (left) and CD8 (right) T cells. (D) CAR+ % over total T cells. (E) Activation and exhaustion markers of CD4+ CAR-T cells. (F) CAR-T cytotoxicity on three different AML cell lines expressing different levels of CD33 (MFI of stained cells on left panel) evaluated via luciferase activity. (G) Cytokine expression in the supernatant of 1:1 E:T co-cultures: IL2 (left) and IFNγ (right). *n* = 6 independent healthy donors aged between 18 and 26 years old *n* = 4 for Nb1 (not included in the first experiments). Statistical analysis was performed using two-way ANOVA for repeated measurements with Tukey's multiple comparisons with FDR correction. Results are shown as mean and error bars represent the standard deviation (SD) derived from biological (A–F) or technical (G) replicates. ∗*p* < 0.05,∗∗*p* < 0.01, ∗∗∗*p* < 0.001.

CAR-T cells generated based on the selected SdAb (Nb1, Nb3, and Nb16) were phenotypically and functionally characterized across a total of 10 independent CAR-T cell productions from healthy donors (Figure 4). For one of the donors, five SdAb-based CAR-T cell preparations were tested (Figure S4). Overall, all SdAb-based CAR-T cells exhibited similar proliferation rates, with no significant differences compared with control ScFv-based CAR-T cells (Figure 4A). The CD4/CD8 ratio was evenly distributed among T cell samples, as well as the subtypes of T cells (Figures 4B and 4C). Transduction efficiencies were also comparable, with CAR+ percentages ranging from 50% to 87% (Figure 4D) and similar CAR densities, with APC median fluorescence intensities ranging from 17,000 to 22,000 (Figure S5B).

CAR-T phenotypes closely resembled those of ScFv-based CAR-T in all cases (Figure 4C). On average, the T cells comprised 75%–80% CD4+ cells and 20%–25% CD8+ T cells. Both CD4+ and CD8+ T cell subpopulations were primarily composed of effector memory cells, followed by central memory cells and effector cells, with very few stem cell memory cells compared with untransduced T cells. Naive T cells were highly represented only in the basal phenotype (day 0, before activation and expansion). The activation marker ICOS was expressed in >90% of CD4+ and CD8+ CAR-T cells, while HLA-DR and CD69 expression varied between 10% and 40% in all CAR-T cells and untransduced T cells. Very low percentages of T cells expressed the intrinsic 4-1BB activation marker. Exhaustion markers LAG3, PD1, and TIGIT were minimally expressed, although TIM3 was present in 60%–70% of CD4+ T cells and 80%–90% of CD8+ T cells (Figures 4E and S5A).

Next, we examined CAR-T cytotoxicity using three AML cell lines expressing different levels of CD33 antigen: HL60, MOLM13, and MV411 (Figure 4F). No significant differences were observed in cytotoxicity between ScFv-based and SdAb-based CAR-T cells at any effector-to-target (E:T) ratios. An increase in cytotoxicity was noted at low E:T ratios in AML cells with higher expression of CD33. When examining cytokine concentrations in the supernatants of CAR-T cells co-cultured with AML cell lines (1:1 E:T ratio), SdAb-based CAR-T cells, particularly Nb3 and Nb16, exhibited significantly higher IL-2 and IFN-γ production compared with ScFv CAR-T (Figure 4G). Cytotoxicity and cytokine production from ScFv-, Nb3-, and Nb16-CAR-T cells were also assessed in co-culture with the control MM1S (CD33-) cell line as a measure of specificity, resulting in poor toxicities against MM1S cells, very low secretion of IFN-γ, and undetectable levels of IL2 (Figure S6).

**Figure 5:**
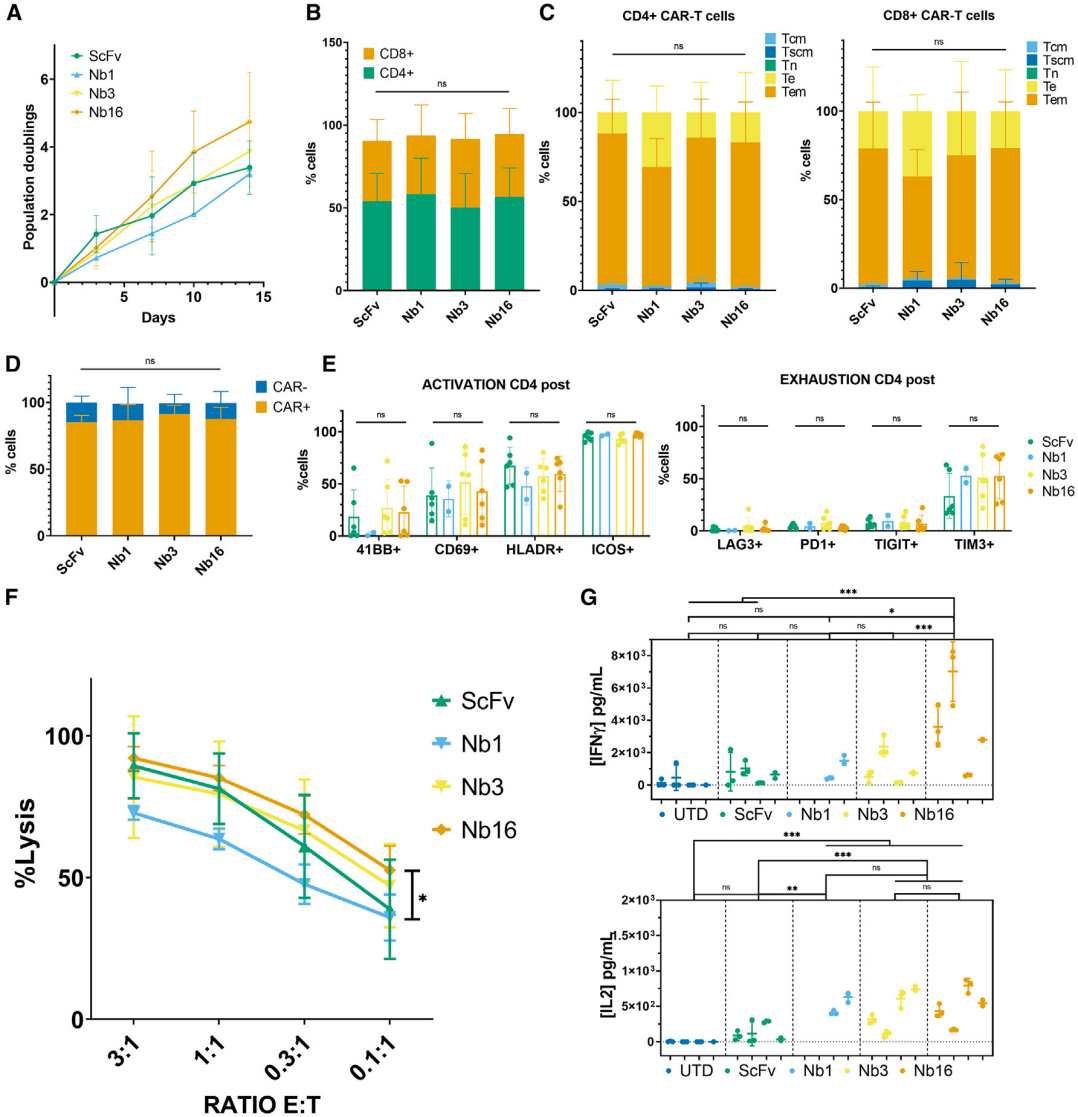
Functional characterization of CAR-T cells after continued stimulation (A) Population doublings during 14 days of CAR-T cell continued stimulation. (B–E) Phenotypic characterization of CAR-T cells via flow cytometry. Repeated measures ANOVA with Tukey’s multiple comparisons (FDR correction) was performed for all compositional analyses. (B) T cell CD8/CD4 populations and (C) subpopulations of CD4 (left) and CD8 (right) T cells. (D) CAR+ % over total T cells. (E) Activation and exhaustion markers of CD4+ CAR-T cells (for CD8+ T cells see Figure S5A). (F) CAR-T cytotoxicity on MOLM13 cells after continued stimulation. Statistical analysis was performed using multiple T tests with two-stage linear step-up procedure of Benjamini, Krieger and Yekutieli ∗*p* < 0.05 (G) Cytokine expression in the supernatant of 1:1 E:T co-cultures: IL-2 (bottom) and IFNγ (top). *n* = 4 independent healthy donors aged between 18 and 26 years old, *n* = 2 for Nb1 (not included in the first experiments). Statistical comparison between groups was analyzed using two-way ANOVA for repeated measurements with Tukey's multiple comparison (with FDR correction for multiple testing). Results are shown as mean and error bars represent the standard deviation (SD) derived from biological (A–F) or technical (G) replicates. ∗*p* < 0.05,∗∗*p* < 0.01, ∗∗∗*p* < 0.001.

To evaluate the long-term functionality of the SdAb-based CAR-T cells, a stress test involving repeated stimulation was conducted. MOLM13 cells were added at a 1:1 E:T ratio every 2–3 days for 2 weeks (Figure S7). SdAb- and ScFv-based CAR-T cells exposed to repeated stimulation did not show differences in proliferative capacity, percentages of CAR+ cells, T cell subpopulations, or markers of activation and exhaustion (Figures 5A–5D). As expected, chronic stimulation of CAR-T cells was associated with higher proportions of T-effector memory and T-effector cells in both CD4 and CD8 populations compared with unstimulated CAR-T cells (Figures 5C and 4C). Repeated stimulation had a very limited impact on activation and exhaustion markers (Figure 5E). Overall, cytotoxicity of CAR-T cells against MOLM13 was comparable to that observed after a single exposure (Figure 5F). Interestingly, at lower E:T ratios, a statistically significant difference in cytotoxicity was observed between ScFv-based CAR-T cells and the Nb16. Consistent with results from single exposure, SdAb-based CAR-T cells (particularly Nb16) exhibited significantly higher IL-2 and IFN-γ levels (Figure 5G).

Overall, our *in vitro* studies indicate that SdAb-based CAR-T cells have a similar phenotype and activity compared with ScFv-based CAR-T cells. However, differences in cytokine secretion were noted both after single and repeated stimulation with AML cells.

### *In vivo* efficacy of SdAb-CAR-T cells in an AML xenograft mouse model

**Figure 6:**
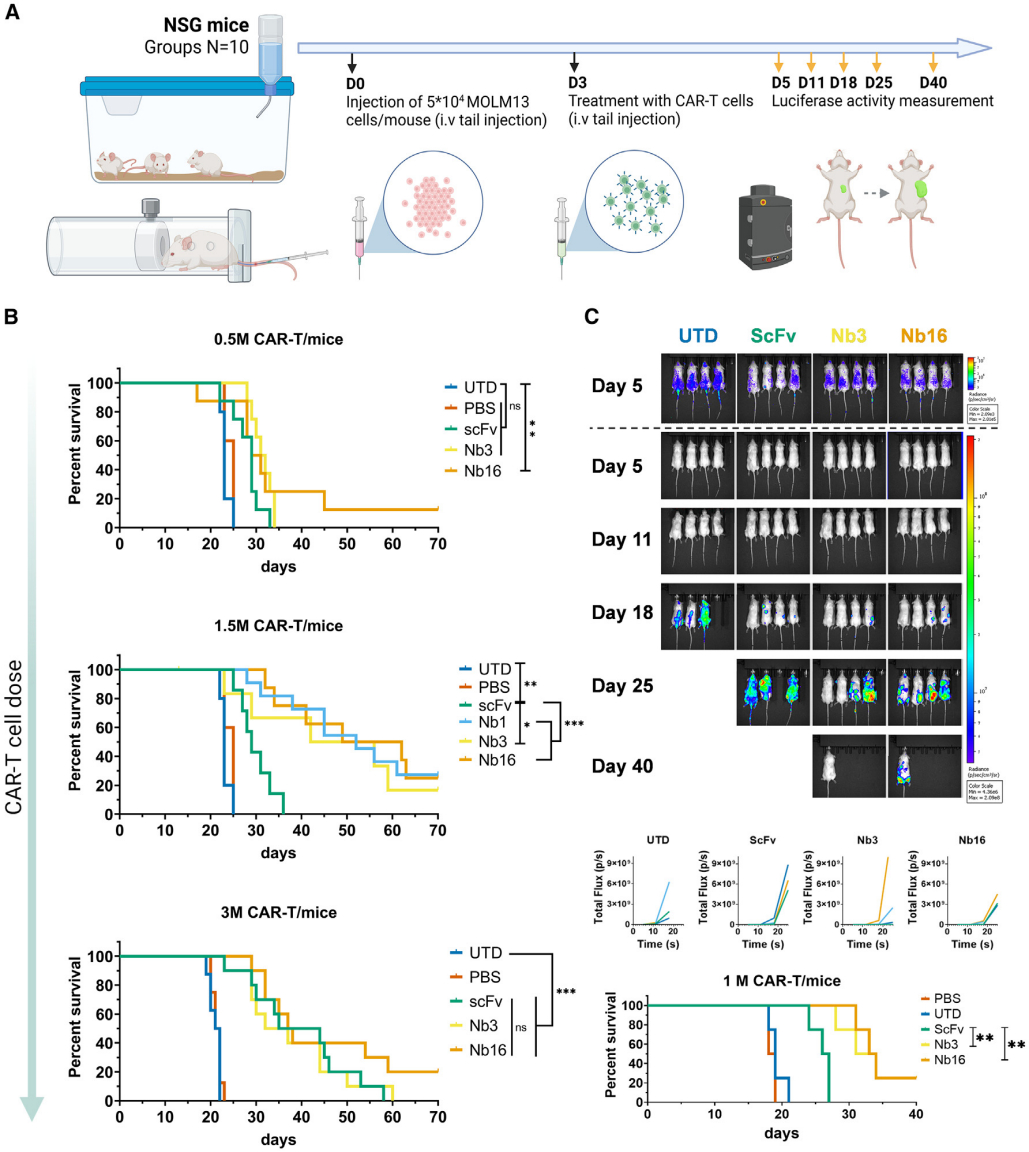
*In vivo* evaluation of CD33 targeted SdAb-based CAR-T cells on mouse xenograft AML model (A) Schematic representation of the optimized procedure on NGS mice created with BioRender.com. (B) Kaplan-Meier survival curves of low-dose (0.5 × 10^6^ CAR-T cells/mice), intermediate-dose (1.5 × 10^6^ CAR-T cells/mice), and high-dose (3 × 10^6^ CAR-T cells/mice) treatments of AML (MOLM13) xenografted mice (*n* = 8). For statistical analysis, survival curves were compared using the Log rank (Mantel-Cox) test, ∗∗*p* < 0.01, ∗∗∗*p* < 0.001. (C) *In vivo* tumoral progression in AML (MOLM13 luciferase+) xenografted mice treated with SdAb/ScFv-based CAR-T cells, and UTD T cells. Top panel: luminescence images of a sample of the treated mice (two males on the left and two females on the right), injected with luciferin at different time points. Bottom panel: progression of MOLM13 cells on each mouse, measured as luciferase activity (Total Flux), and corresponding survival curves, analyzed using the Log rank (Mantel-Cox) test, ∗∗*p* < 0.01, ∗∗∗*p* < 0.001.

Finally, we evaluated the *in vivo* functionality of SdAb-based CAR-T cells in an AML xenograft NSG mouse model (Figure 6A). Mice were infused with 5 × 10^4^ MOLM13 cells on day 0, and CAR-T cells were infused on day 3. Three different doses of CAR-T cells (0.5 × 10^6^, 1.5 × 10^6^, and 3.0 × 10^6^) were assessed (Figure 6B). Our AML model presented a dose-dependent response to CAR-T cell treatment and a critical dose to observe differences in survival. At the lower dose (0.5 × 10^6^ CAR-T cells/mice), efficacy was limited, and only a statistically significant increase in survival was observed for Nb16-based CAR-T cell-treated mice. In contrast, when animals were treated with 3 × 10^6^ CAR-T cells, both SdAb- and ScFv-based CAR-T cells were associated with prolonged survival without significant differences between the constructs, suggesting a threshold effect for therapeutic efficacy in this model. Finally, when we analyzed animals receiving a dose of 1.5 × 10^6^ CAR-T cells per mouse, we observed a significant increase in survival in those receiving the Nb16-based CAR-T cells compared with those receiving ScFv-CAR-T or other SdAbs. These results were consistent with the tumor load observations using luciferase assays (Figure 6C).

To determine whether a different co-stimulatory signal could enhance the efficacy of Nb16-CAR-T cells, we compared Nb16-CAR cells harboring CD28 or 4-1BB as co-stimulatory domains (Nb1628z or Nb16BBz) (Figure S8). *In vitro*, these constructs showed similar cytotoxicity at a 3:1 E:T ratio but differed in cytokine secretion levels, with higher IFN-γ and IL2 production observed in constructs with 4-1BB as the co-stimulatory domain. *In vivo*, no significant differences in survival were observed between animals with AML xenografts treated with these constructs. The characterization of CAR-T cells *in vivo* included an evaluation of their immunophenotypes, proliferation, and persistence in treated mice, as described in the materials and methods section and summarized in Figure S9A. No notable differences were detected in the immunophenotypes of CAR-T cells extracted from treated mice, 3 and 7 days post-treatment (Figure S9B). However, an increase in CAR-T cell counts was observed in mice treated with Nb16 and Nb3 CAR-T cells on day 7, suggesting enhanced proliferation and persistence (Figure S9C).

## Discussion

SdAbs are versatile tools with attractive properties for CAR-T cell development. Their small size (12–15 kDa) together with their monomeric nature, reduces the number of potentially immunogenic epitopes and minimizes the risk of aggregation.^14^ Llama-derived VHH domains share ∼95% sequence identity with human IGHV3 germline genes,^15^ comparable to that of humanized murine VH domains, such as trastuzumab. Over 35 clinical trials involving SdAb-based biologics have demonstrated low immunogenicity.^16^ This promising record contributes to the advantages of using SdAbs in multiple therapeutic and diagnosis approaches in oncology.^17^

In contrast, ScFvs, particularly those derived from murine sources, carry a higher risk of immunogenicity due to their larger size (∼27–30 kDa) and the presence of non-human epitopes. Tonic signaling in ScFv-based CARs can arise from dimeric arrangements in the VH and VL interface, such as VH-VH aggregation or VH-VL mispairing.^18^^,^^19^ In contrast, SdAbs, being monomeric in nature, are less susceptible to such undesired interactions.^6^^,^^10^

The results of our study demonstrate the potential of SdAbs for generating CAR-T cells, targeting CD33, commonly expressed in AML. Our findings are consistent with previous efforts to develop SdAbs against CD33, using similar procedures for radioisotope-conjugate imaging and bi-specific T cell engagers (BiTEs).^20^^,^^21^ Binding affinity to the target antigen is widely recognized as a crucial factor in CAR-T cell activation and effector function.^7^^,^^22^^,^^23^^,^^24^ However, it is important to note that CAR-T cell performance is influenced by various factors beyond binding affinity, including the nature of the tumor (solid or hematological), the avidity of interaction in the context of co-stimulation, and the specific epitope recognized by the CAR.^22^^,^^25^^,^^26^^,^^27^ Therefore, having a diverse range of SdAbs with varying affinities, such as those developed in this work, is highly advantageous for optimizing CAR-T cell therapies. The reference ScFv affinity was higher than that of any of the SdAbs. Nevertheless, higher affinity is not always optimal: CAR-T cells with moderate affinities (nanomolar range), have proven better performances, preventing exhaustion, toxicity, antigen-induced cell death and antigen trogocytosis while increasing expansion and persistance.^22^^,^^28^^,^^29^

Epitope location also plays a significant role in CAR-T cell efficacy, as it determines the synaptic distance in conjunction with the CAR hinge length.^30^^,^^31^ Our epitope competition assays revealed partial or complete competition for antigen binding among the evaluated SdAbs, indicating the presence of epitope overlap, steric hindrance, or conformational changes in the antigen that prevent dual binding. For example, the binding of Nb1 and Nb16 completely inhibited the binding of Nb3 and Nb17, suggesting epitope overlap. Partial competition was observed between Nb8 and Nb1, or between Nb8 and Nb16, but only when Nb8 was bound first to CD33. These findings suggest that some of the tested SdAbs may share binding sites on CD33, and functional differences between the respective CARs may rely on variations in binding affinity. The reference ScFv did not compete for CD33 binding with any of the candidate SdAbs, indicating that it does not share an epitope location with them.

To further elucidate the binding characteristics and epitope locations of the SdAbs, in addition to *in silico* structural predictions tools, studies such as epitope mapping techniques or structural studies of the SdAb-CD33 complex using NMR or X-ray crystallography should be conducted.^32^^,^^33^ These studies would provide valuable insights into whether the SdAbs bind to the proximal IgC or distal IgV domains in CD33.^34^ Previous studies have reported that binding to proximal CD33 epitopes enhances functionality compared with distal ones.^31^ Furthermore, deepening our understanding of these interactions could be instrumental in designing CARs based on SdAbs, potentially leading to the development of tandem CARs similar to the Food and Drug Administration (FDA)-approved anti-BCMA CAR-T therapy Cilta-cel.^35^ Additionally, this knowledge could inform the development of bi-specific CARs targeting other AML targets, such as CLL1 or CD123.^36^^,^^37^^,^^38^

Our results align with other studies that leverage this property by incorporating SdAbs as CAR recognition moieties.^10^^,^^11^ Thus, previous reports evaluate SdAb-based CARs specifically targeting CD33 in AML.^39^^,^^40^ De Munter et al. demonstrated robust *in vitro* cytotoxicity and cytokine production using SdAb-based second-generation CAR-T cells against CD33, although tonic signaling was not evaluated. Moreover, Appelbaum et al., using a dimerizing agent regulated immunoreceptor complex (DARIC) in a split SdAb-CAR, found that the SdAb targeting the C2 domain of CD33 had a K_D_ of 2.2 nM, similar to our Nb16 (4.18 nM). The promising results obtained using this SC-DARIC33 led to a phase I clinical trial for pediatric R/R AML named PLAT-08 (NCT05105152),^41^ although the trial was finally held by the FDA due to fatal adverse effects.^42^ Finally, Schneider et al. generated human immunoglobulin VH domain-only based CAR-T cells targeting CD33 demonstrating promising preclinical results,^43^ which further progressed to clinical trials (VCAR33, NCT05984199).^44^ Overall, developing optimized CAR-T cells to effectively and safely treat AML is still challenging. In our work, we developed various SdAb-based CAR-T cells with lower tonic signaling and a wide range of affinities that could potentially reduce off-target activation and improve general CAR-T cell performance.^45^^,^^46^

We found that most of the SdAb-based CARs exhibited lower tonic signaling compared with the reference ScFv-based CAR, maintaining antigen-dependent activation. This property could potentially mitigate early T cell exhaustion and enhance T cell persistence.^47^ Furthermore, we investigated the relationship between SdAb affinity and tonic signaling. Interestingly, we found that SdAb affinity does not strictly correlate with tonic signaling. For example, Nb3 and Nb16, which have overlapping epitopes on CD33 but significantly different affinities, demonstrated similar activation profiles. On the other hand, Nb8, which recognizes a different epitope from Nb3 and Nb16, exhibited higher tonic signaling despite having a binding affinity between that of the other SdAbs. Based on these findings, Nb8 was deemed unsuitable as a CAR candidate due to the potential risk of increased off-target toxicities. In contrast, Nb16, Nb17, Nb3, and Nb1 CARs demonstrated lower ligand-independent tonic signaling in the Jurkat TRP system, indicating a promising attribute. Overall, the lower tonic signaling observed in SdAb-based CAR constructs represents a significant advancement in the field of CAR-T cell therapy, by potentially enhancing T cell persistence and reducing off-target toxicities, thereby improving the safety and efficacy of CAR-T cell treatments.

Finally, our study demonstrates that these novel CAR-T cells based on SdAbs, particularly Nb1, Nb3, and Nb16, exhibit robust antitumoral efficacy. Despite the similar *in vitro* performance among the SdAb-based CAR-T cell candidates, the affinity differences suggest that factors other than binding strength, such as epitope proximity, might influence their functional outcomes. Flow cytometry analysis revealed that SdAb-based CAR-T cells share phenotypic characteristics with traditional ScFv-based CAR-T cells, encompassing T cell subpopulations and markers of activation, exhaustion, and cytotoxicity. However, a critical distinction was observed in cytokine production: Nb3 and Nb16 CAR-T cells produced notably higher levels of IL-2 and IFNγ upon activation. This cytokine profile is pivotal, as it can enhance T cell proliferation and persistence,^48^ while posing risks of cytokine release syndrome (CRS) if not carefully managed.^49^^,^^50^

Repeated stimulation assays confirmed that SdAb-based CAR-T cells maintain a stable activated phenotype, orchestrating a prolonged proinflammatory and proliferative immune response. This characteristic likely contributes to enhanced T cell persistence and antitumoral activity over the long term.^51^ *In vivo* experiments further validated the therapeutic potential of SdAb-based CAR-T cells, showing comparable efficacy to the reference ScFv-based CAR-T cells, particularly at lower doses. Notably, these treatments extended mouse survival and significantly delayed tumor progression, highlighting the potential of SdAb-based CAR-T cells, especially Nb16, as promising therapeutic agents against AML with lower therapeutic indexes, that may ease CAR-T manufacturing and overcome adverse effects.

The mechanisms underlying the improved performance of SdAb-based CAR-T cells remain to be fully elucidated. We hypothesize that reduced CAR tonic signaling and increased IL-2 production may play crucial roles by enhancing persistence. However, the immunosuppressed mouse xenograft model used in our study does not allow for a comprehensive evaluation of potential adverse effects, such as CRS and neurotoxicity, associated with elevated cytokine levels. To address these safety concerns, future studies should employ humanized immunocompetent mouse models and consider murine CAR-T cell production. Additionally, it is imperative to assess SdAb cross reactivity with human tissues to prevent off-target toxicities before advancing these therapies to clinical trials.

In conclusion, SdAb-based CAR-T cells emerge as a promising innovation in CAR-T cell therapy, offering distinct properties over a conventional ScFv-based construct. Their lower propensity for high tonic signaling and enhanced cytokine production upon activation could potentially overcome current manufacturing limitations by requiring fewer cells for effective therapy. Further research is essential to validate these findings and explore the clinical potential of SdAb-based CAR-T cells in AML treatment, with the ultimate goal of developing safer, less toxic, and more efficient therapeutic options.

## Materials and methods

### Generation of the immune SdAb library

A male llama was immunized with the CD33 recombinant ectodomain (SinoBiological #12238-H08H) four times in-between 20 and 30 days at doses of 150 and 200 μg, alongside other antigens (SLAMF7, CD70 and EDA) using Freund’s adjuvants, while monitoring anti-CD33 humoral immune response by indirect enzyme-linked immunosorbent assay (ELISA), using CD33-coated plates, plasma samples, and the Goat anti-Llama IgG (H + L) Secondary Antibody, horseradish peroxidase (HRP) (Thermo Fisher Scientific Cat# A16060, RRID:AB_2534733). Peripheral blood mononuclear cells (PBMCs) were isolated from peripheral blood by Ficoll density gradient and total RNA was extracted with standard TRIzol-chloroform method, evaluating its quality by observing the 28S and 18S ribosomal RNA on agarose gel electrophoresis. cDNA was generated using the RevertAid first-strand cDNA synthesis kit (Thermo Scientific #K1621), and SdAb DNA was obtained by PCR amplification using VH1, VH3, and VH4 forward primers and JH reverse primer (Table S1) with the Kapa TAQ kit (Roche #KK1015). The SdAb DNA repertoire was digested with SfiI enzyme (NEB #R0123L) and ligated into SfiI digested and rSAP (NEB# M0371L) dephosphorylated pCOMB3XSS phagemid vector (RRID:Addgene_63890) using T4 DNA ligase (Thermo Scientific #EL0012) in a 1:3 ratio of vector to insert. Electrocompetent *E. coli* TG1 (LGC #60502-2) and ER2738 (LCG #60522-2) were transformed with the ligation products, recovered in SOC medium and selected with ampicillin before being infected with M13KO7 helper phage (NEB #N0315), followed by selection with kanamycin. Phages were precipitated from the overnight culture supernatant using PEG8000-NaCl double precipitation. The final phage libraries were resuspended in PBS with 1% BSA and titrated by infecting *E. coli* ER2378 and plate counting.

### Identification and isolation of CD33-Binding SdAbs

To select CD33-specific binders, panning rounds were performed in 96-well high-binding EIA plates (Corning #3590) coated with 1 μg/well of CD33. After blocking with 1% BSA in PBS, a total of 2 × 10^11^ PFU of phage libraries were first cleared in uncoated BSA-blocked wells ("pre-panning" step) to eliminate potential binders to BSA or to the plate surface. Cleared phages were incubated in CD33-coated and BSA-blocked wells for 1 h at room temperature and washed 25 times with PBS 0.05% Tween 20. Binders were eluted using 10 mg/mL bovine pancreatic trypsin (Sigma #T4799256). Protease inhibitor cocktail (Cell Signaling #5871S) was added to the output phages, and ER2738 cells were infected for titration and phage amplification using helper phage M13KO7. In subsequent rounds, the amplified output phages (AO) were used as input, maintaining the input at 2 × 10^11^ pfu so that the output/input phage ratio reflects phage enrichment. Phage ELISA was performed by adding 2 × 10^11^ pfu of each output and the original library to CD33-coated plates and incubating with anti-M13 (HRP) antibody (RRID:AB_2857928). Screening of positive clones was conducted from two sources: the phagemid containing isolated clones (pCOMB3XSS) and the further subcloned clones containing the SdAbs in an expression vector derivative from pET-28a(+) RRID:Addgene_141289 named pETMod, containing the OmpA periplasmatic localization signal peptide. Single colonies from the titration plates of outputs 2 and 3 were grown and induced with 2 mM IPTG for SdAb expression, which typically leaks SdAb into the culture supernatant further tested individually in CD33 binding ELISA using Anti-HA HRP (Roche Cat# 11667475001, RRID:AB_514509). Once enrichment reached more than 80% of positive clones, the SdAb repertoire of the last output was amplified in *E. coli*, and phagemid DNA extraction was performed using the Qiaprep plasmid kit (Qiagen #27106). The enriched SdAb repertoire was subcloned into the pETMod and further sequenced using the Sanger method with the ompseq forward primer (Table S1).

### Expression and purification of anti-CD33 SdAbs

Sequences of the positive clones were aligned using Clustal Omega (RRID:SCR_001591) in Jalview (RRID:SCR_006459), where a phylogenetic tree was constructed (BLOSUM62 matrix). SdAb candidates representing each cluster/family were selected for production and characterization, under the assumption that similar CDR sequences often interact with the same epitope in the antigen, while different ones are more likely to bind different ones. Candidate SdAbs were produced by culturing the transformed *E. coli* expression strains (Novagen # 69450-M, Agilent # 230245). Bacterial periplasmic lysis was carried out using RIPA buffer (Sigma #R0278) supplemented with cOmplete Mini EDTA-Free protease inhibitor cocktail (Roche #11836170001) and DNAseI (Roche #10104159001). The lysate was centrifuged at high speed (15,000 × *g* for 30 min at 4°C) to remove cell debris and supernatant was filtered through a 0.2-μm syringe filter and purified using Äkta Pure FPLC (Cytiva). For the first purification step, HiTrap Chelating 5 mL columns (Cytiva #17040903) were used for ion metal affinity chromatography (IMAC) purification (nickel charged). And the second purification step included ion-exchange chromatography (IEC) in RESOURCE Q/S columns (Cytiva #17117701 #17117801), depending on the isoelectric point of each SdAb. Purified SdAbs were dialyzed in PBS overnight, and protein concentration was assessed using the Pierce BCA Kit (Thermo Scientific #23225). Reference ScFv “My96” production and purification was outsourced to GenScript using the TurboCHO cell-free expression system.

### Binding characterization of CD33-targeting SdAbs

ELISA was performed to determine relative affinities (EC_50_) to CD33 using Anti-HA HRP (Roche Cat# 11667475001, RRID:AB_514509) for the SdAbs and Biotin-SP Anti-Mouse F(ab’)_2_ (Jackson Immunoresearch #115-066-006, RRID: AB_2338558) a Streptavidin HRP (BD #554066, RRID:AB_2868972) for the ScFv. SPR, was conducted to determine thermodynamic and kinetic constants, on the Biacore X100 device (Cytiva) using NTA sensor chip (Cytiva #BR100034, Lot.10335787), following single-cycle kinetic (SCK) procedures. The sensor chip was charged with nickel solution and disposed of His-tagged CD33 ectodomain on flow cell 2, resulting in a non-covalent attachment for the protein (C-terminal). Consecutively, EDC/NHS reagents were added to cross-link the CD33-oriented ectodomain covalently to the chip surface, and nickel was stripped with EDTA solution to eliminate non-covalently bound protein. Flow Cell 1 was used as a reference, following the same procedure but without adding CD33; 388 RUs of CD33 were finally immobilized covalently. The working buffer was HBS-EP+ to avoid metal ions from chelating on the NTA surface, given that SdAbs also have histidine tags. Serial dilutions of the five candidates SdAbs (Nb1, Nb3, Nb8, Nb16, and Nb17) were made: 1,000, 333, 111, 37.0, 12.3 nM, and for the My96 ScFv the concentrations were 50, 25, 12.5, 6.25, and 3.12 nM. For each SdAb, three startup cycles were performed before four sample cycles: the first and last cycles with 0 mM of sample (baseline controls) and duplicate cycles in the middle with the five concentrations. The sample flow was set at 10 μL/min, with a contact time of 300 s and a dissociation time of 600 s. Chip regeneration was performed using 10 mM glycine at pH 2 three times between cycles (contact 30 s, at 30 mL/min flow). For the ScFv, shorter contact times were set to enable the chip regeneration in mild conditions (120 s). SCK curves were fitted to a 1:1 binding model to determine the rate constants of association: kon (M-1s-1), and dissociation: koff (s-1), and the global dissociation constant: KD (KD (M) = koff/kon) that indicates the binding affinity. Fitting was considered good when tc > 100 × ka, U value >25, and Chi2 < 10% Rmax, according to manufacturer instructions. Tc: Flow rate-independent component of the mass transfer constant, ka: Association rate constant (M-1s-1), U value: estimate of the uniqueness of the calculated values for rate constants and Rmax, Chi2: closeness of fit, calculated as the average squared residual, Rmax: Analyte binding capacity of the surface (RU). SdAb competition was evaluated in epitope binning assays by BLI using Octet high-precision Streptavidin 2.0 SAX2 sensors (Sartorius #18–5136) in the Octet N1 device. Biotinylated CD33 (bio-CD33) was immobilized at a concentration of 100 nM, resulting in a signal increase of 1.5–2 nm. The concentration of the first antibody was fixed at 400 nM and for the secondary binding a mixture of 400 nM of the first antibody and 200 nM of the second one was prepared. Cycle steps were as follows: (1) 30 s initial baseline (PBS), (2) 300 s of bio-CD33 immobilization, (3) 30 s of pre-binding baseline (PBS), (4) 600 s of first SdAb binding, (5) 400s of second SdAb binding. In this work, out of the five SdAb candidates, the three exhibiting the lowest koff (Nb1, Nb8, and Nb16) were used as the initial-binding antibodies in an in-tandem binning approach. The five SdAb candidates were utilized as second-binding antibodies in all instances. Aligned curves were graphed using GraphPad Prism 8.0 (RRID:SCR_002798).^52^ SdAb binding to AML cell lines was evaluated using MOLM13 cells (RRID:CVCL_2119) stained with each SdAb (HA-tagged) and PE anti-HA.11 Epitope Tag (BioLegend Cat# 901518, RRID:AB_2629623) and acquired in Cytoflex Dx Flex flow cytometer to determine CD33 binding. For reference, BV510 anti-CD33 (BioLegend Cat# 303421, RRID:AB_2565657) (clone WM53) was used alongside BV510 Mouse IgG1κ isotype control (BioLegend Cat# 400172, RRID:AB_2714004). SdAbs were used as the primary antibody at a concentration of 1 μg/mL and incubated for 30 min at room temperature. An irrelevant SdAb was used as the isotype control in this step. After washing with FACS buffer, a 1/100 dilution of anti-HA PE-conjugated antibody (BioLegend # 901517) was used as the secondary antibody, incubating for 15 min at room temperature. FlowJo (RRID:SCR_008520) was used to analyze data and generate graphical figures.

### Second-generation CAR design and lentiviral vector production

The CAR design utilized a second-generation CAR scaffold, incorporating the EGF1a promoter, CD8 signal peptide, CD8a hinge and transmembrane domain, 41BB (CD137) co-stimulatory domain, and CD3ζ signaling domain within a third-generation self-inactivating lentiviral vector, derivative form pCCL (RRID:Addgene_132429). Truncated epidermal growth factor receptor (EGFRt) was included as the reporter molecule in most cases, though mCherry and blue fluorescent protein (BFP) were used in some instances. SdAb sequences were inserted in the place of the ScFv binding domain when required. The reference ScFv used in this work was based on anti-CD33 My96 clone.^53^^,^^54^ All constructs were synthesized by GenScript Biotech. Lentiviral vectors were produced in HEK293T cells (RRID:CVCL_0063) using established protocols that use packaging vectors pMDLg/pRRE (RRID:Addgene_12251), pRSV-Rev (RRID:Addgene_12253) and pMD2.G (RRID:Addgene_12259)^55^ and titrated by infecting HEK293T cells in 24-well culture plates (100,000 cells/well) with serial dilutions of the concentrated lentivirus. The EGFRt+ (CAR+) population was then measured by EGFRt staining with APC anti-EGFRt (BioLegend Cat# 352906, RRID:AB_11150410) and flow cytometry.

### Activation profile in Jurkat-TPR system

Jurkat triple parameter reporter cells (Jurkat-TPR) derived from J76, a TCR alpha and beta chains knockout derived from Jurkat E6.1 cell line (RRID:CVCL_0367), were kindly provided by P. Steinberg, Medical University of Vienna. These modified cells facilitate the report of three main CAR activation signaling pathways: nuclear factor of activated T cells (NFAT), nuclear factor kappa B (NF-κB), and activator protein 1 (AP1) by producing green fluorescent protein (GFP), BFP, or mCherry (mCh), respectively.^56^ Jurkat-TPR cells were transduced with lentivirus harboring the five SdAb-based and the ScFv-based CARs and further co-cultured in triplicate alongside CD33-expressing MOLM13 cells (RRID:CVCL_2119) and an unstimulated control (culture media), to evaluate tonic signaling. Untransduced (UTD) Jurkat-TPR cells served for baseline fluorescence levels and as negative controls in the absence and presence of MOLM13 cells. CAR expression was determined using APC anti-EGFRt (BioLegend Cat# 352906, RRID:AB_11150410) antibody (clone AY13). Activation of the NFAT, NF-κB, and AP1 pathways was assessed before and 24 h after co-culture with tumor cells using the CytoFLEX LX Flow Cytometer (Beckman Coulter) and analyzed in CytExpert Software (RRID:SCR_017217).

### CAR-T cell production

Peripheral blood was extracted from a total of 11 healthy donors listed at the Clínica Universidad de Navarra (three male and eight female). Blood was diluted with PBS and carefully layered on centrifugation tubes containing Ficoll for density gradient centrifugation (400 × *g*, 30 min with no deceleration). PBMC halos were collected, washed with PBS and counted using Nexcelom chambers (Revvity #CHT4-SD100) and Cellometer K2 (Nexcelom, Revvity). For T cell isolation, magnetic anti-CD4 and anti-CD8 Microbeads (Miltenyi Biotec Cat# 130-045-101, RRID:AB_2889919 and Miltenyi Biotec Cat# 130-045-201, RRID:AB_2889920) were added following providers’ instructions. Magnetic automatic cell sorting (MACS) was performed using the autoMACS Pro Separator (Miltenyi Biotec). T cells (CD4+ and CD8+) were then activated using T cell TransAct (Miltenyi #130-111-160) beads and cultured at a concentration of 1 million cells/mL in RPMI medium supplemented with 3% human serum (Sigma #H4522), 1% penicillin/streptomycin, 625 IU/mL IL7, and 85 IU/mL IL15 to maintain the stem-like phenotype. Forty-eight hours later, T cells were infected with lentivirus at an MOI of 3 with 10 μL/mL of LentiBOOST (Sirion Biotech). On day 5, cells were centrifuged and resuspended in new RPMI complete medium with IL7 and IL15 maintaining a concentration of 1 million cells per mL until day 12–14. The percentage of CAR expression was assessed on day 7–9 of production using APC anti-EGFRt (BioLegend Cat# 352906, RRID:AB_11150410) and performing flow cytometry analysis using Cytoflex Dx and CytExpert Software (RRID:SCR_017217).

### CAR-T phenotypical and functional characterization

The initial T cell and final CAR-T cell (day 12–14) subpopulations, activation, and exhaustion phenotypes were characterized using five different antibody panels (Table S2) in flow cytometry assays using FACS Canto II (BD Bioscience) device and analyzing results in FlowJo (RRID:SCR_008520). To assess CAR-T cell cytotoxicity, GFPLuc+ MOLM13 (RRID:CVCL_2119), MV411 (RRID:CVCL_0064), and HL60 (RRID:CVCL_0002) AML cell lines were co-cultured with T cells in a 96-well U-bottom culture plate using 10,000 tumoral cells and effector:tumor ratios of 3:1, 1:1, 0.3:1, and 0.1:1 in complete RPMI medium. CD33 negative MM1S (RRID:CVCL_8792) cell line was used as control. After 24 h, cells were centrifuged, and the supernatant was collected for cytokine assessment. Pellets containing CAR-T cells and potentially lysed tumoral cells were washed with PBS, resuspended in 30 μL of fresh RPMI medium, and transferred to a Nunclon plates (Thermo Scientific #10072151). Then, 30 μL of Bright-Glo reagent (Promega #E2610) was added to each well, and after 5 min, bioluminescence was measured using a GloMax Discover device (Promega). IFN-γ, TNF-α, and IL-2 concentrations in the co-culture supernatants (1:1 and 1:0.3 ratios) from the cytotoxicity tests were assessed using OptiEIA ELISA kits (BD #555142, #555190, #555212) following the provider’s instructions. Additionally, we used an Nb16-CAR construct harboring mCherry reporter protein to transduce T cells and visualize cytotoxic activity over GFP+ MOLM13 cells in fluorescence microscopy (Figure S10). For continued re-stimulation, 4 million of CAR-T cells were co-cultured at a 1:1 Effector:Target (E:T) ratio with MOLM13 cells, totaling four challenges over 2 weeks (every 2–3 days). Cell counting and %CAR indicated the amount of MOLM13 cells necessary to achieve a 1:1 E:T ratio, at 1 million cells/mL in complete RPMI. New cytotoxicity assays and final phenotype staining for flow cytometry were performed after the 2 weeks of re-stimulation.

### Cell lines and culture conditions

HEK293T cells (RRID:CVCL_0063) were cultured in Dulbecco’s modified Eagle’s medium (Gibco #41966-029), 10% FBS, 1% penicillin/streptomycin. MOLM13 cells (RRID:CVCL_2119), MV411 cells (RRID:CVCL_0064), HL-60 cells (RRID:CVCL_0002), and MM1S (RRID:CVCL_8792) cells were cultured in RPMI (Gibco #61870-010), 10% FBS, 1%penicillin/streptomycin. Jurkat-TPR: modified (triple reporter fluorescent proteins) from J76 derived (TCR alpha and beta chains KO) from Jurkat E6.1 (RRID:CVCL_0367) were cultured in RPMI (Gibco #31870-025), 10% FBS, 1% penicillin/streptomycin, 1% glutamine. All cell lines were checked for mycoplasma contamination periodically with MycoAlert kit (Lonza # LT07-318) and authenticated in the center’s genomics facility.

### *In vivo* MOLM13 NSG mouse xenograft model

To evaluate the preclinical activity of the SdAb-based CAR-T cells, NOD-SCID-Il2rg−/− (NSG) mice (RRID:IMSR_JAX:005557)^57^ aged between 10 and 12 weeks were xenografted with 50,000 MOLM13 (CD33+, luciferase+) cells via intravenous tail injection. Three days after, mice were treated with CAR-T cells or UTD T cells at doses of 3, 1.5, and 0.5 million cells per mouse, and a group treated with PBS was used as control. Sample size was calculated considering a power of 80%, α = 0.05, β = 0.2 and expected mean survival of 20 ± 5 days (post-tumor) for UTD and PBS groups, and at least 27 days for treated mice, resulting in *n* = 8 for each group, four male and four female individuals. Survival was monitored from tumor injection (day 0), animals with loss of mobility were euthanized following ethical considerations. For tumoral progression measurement in a separate assay, luciferase activity was measured in IVIS Spectrum imaging device (Revvity). Animals (*N* = 4, two male, two female) were injected intraperitoneally with 100 μL of luciferin solution (Regis Technologies #1-360222-200) and anesthetized with an inhalation mixture of isoflurane and oxygen. Five to 10 min after luciferin injection, images were captured and saved for further analysis in PerkinElmer IVIS Spectrum *In Vivo* Imaging System (RRID:SCR_018621). To evaluate CAR-T cells *in vivo* persistence and immunophenotypes, mice were xenografted with 50,000 MOLM13 cells and treated with 1.5 million CAR-T cells per mice (groups: ScFv, Nb3, and Nb16). A representative sample composed of two males and two females from each group were euthanized 3 and 7 days after treatments for spleen extraction and isolation of cells by mechanical disruption, ACK buffer treatment (Gibco A1049201) to lyse erythrocytes and centrifugation. In flow cytometry analysis, CD3 and EGFR staining was included in panel 1 (persistence) and TIM3, HLADR, and 41BB staining was performed in panel 2 (activation/exhaustion) together with CD4, CD8, CCR7, CD45Ra, and EGFR staining for CAR-T cell gating. All experimental protocols received approval from the Ethics Committee of the University of Navarra (120/18, 051/22) and the Institute of Public Health of Navarra in compliance with European Council Guidelines. NSG mice were obtained from The Jackson Laboratory (JAX), bred, and housed in a pathogen-free facility within our institution.

### Statistical analysis and data visualization

Statistical analyses and figures for publication were performed and prepared in GraphPad Prism 8.0 (RRID:SCR_002798). Specific tests utilized in this study are specified in the legend of each figure. Schematic figures were created in ©BioRender - biorender.com.

In the supplemental material, a schematic summary of the general processes for SdAb-based CAR-T cell development is illustrated in Figure S1.

## Data and code availability

Data collected in this study are available upon request from the corresponding authors.

## Acknowledgments

This study was supported by the project PID2023-148573OB-I00 financed by MICIU/AEI /10.13039/501100011033 and FEDER, UE; Project PID2020-115875RBB-I00 financed by MICIU/AEI /10.13039/501100011033; Project RTI2018-101708-A-I00 financed by MICIU/AEI /10.13039/501100011033 and FEDER “A way to make Europe”. We thank Instituto de Salud Carlos III co-financed by FEDER “A way to make Europe” (PI19/00922 and PI20/01308), Red de Terapias Avanzadas TERAV (RD21/0017/0009), 10.13039/501100014139Centro de Investigación Biomédica en Red de Cáncer CIBERONC (CB16/12/00489), 10.13039/501100000780European Commission (H2020-JTI- IMI2-2019-18: Contract 945393, T2EVOLVE; SC1-PM-08-2017: Contract 754658), 10.13039/501100000289Cancer Research UK [C355/A26819], FC 10.13039/501100002704AECC and AIRC under the Accelerator Award Program, Fundación La Caixa La Caixa (HR24-01000. iMMprove) and 10.13039/501100017266Gobierno de Navarra, Departamento de Desarrollo Economico y Empresarial (AGATA: 0011-1411-2020-000011 and 0011-1411-2020-000010; DESCARTHeS: 0011-1411-2019-000079 and 0011-1411-2019-000072; DIAMANTE: 0011-1411-2023-000074 and 0011-1411-2023-000105 alloCART-LMA: PC011-012; SOCRATHeS; 0011-1411-2023-000105 and 0011-1411-2023-000074; SYNTHBIOMICS: 0011-1411-2023-000035). L.P.-C. was supported by 10.13039/501100004587Instituto de Salud Carlos III through a Miguel Servet contract (CP22/00005), co-funded by the 10.13039/501100000780European Union. We thank the Fundación Fuentes Dutor and Fundación Kereis 4 Impact for financial support.

## Author contributions

F.B.-B.: Investigation, methodology, conceptualization, data curation, formal analysis, validation, visualization, writing – original draft. E.M.: Investigation, methodology and data curation. V.I., I.I.-S. and F.R.: Investigation and data curation. P.J., S.R.-D., and R.M.-T.: Investigation, methodology and validation. I.A.-B.: Investigation and Software. N.G.-C. and L.P.-C.: Investigation, methodology, and validation. J.J.L. and L.V.: Conceptualization, supervision, validation and methodology. J.R.R.-M., F.P., and A.P.-L.: Conceptualization, supervision, resources, funding acquisition, project administration, writing-review and editing.

## Declaration of interests

A patent application regarding the generation of anti-CD33 SdAbs has been submitted by Fundación para la Investigación Médica Aplicada, Universidad de Navarra, and Nanogrow Biotech Corporation. The authors declare no additional conflicts of interest.

## Supplemental information

Document S1. Figures S1–S10, Tables S1, and S2

Document S2. Article plus supplemental information
