# Supplementary material for: Discovery and preclinical development of a SdAb-based CAR-T technology for targeting CD33 in AML
Source: Mol Ther Oncol. 2025 Feb 11;33(1):200949. doi: 10.1016/j.omton.2025.200949 (PMC11904528; doi:10.1016/j.omton.2025.200949)
Supplement: Document S1. Figures S1–S10, Tables S1, and S2 [file mmc1.pdf]

OMTON, Volume *33*

Supplemental information

Discovery and preclinical development of a SdAb-based CAR-T technology for targeting CD33 in AML

Franco Bernasconi-Bisio, Eva Molina, Vianca Ibarra, Inés Ibáñez-Sala, Federica Rochira, Patricia Jauregui, Saray Rodríguez-Diaz, Rebeca Martínez-Turrillas, Iñigo Azagra-Barber, Nuria Gómez-Cebrián, Juan José Lasarte, Leonor Puchades-Carrasco, Lucía Vanrell, Juan Roberto Rodríguez-Madoz, Felipe Prósper, and Antonio Pineda-Lucena

Table S1: Oligos used for SdAb library construction and SdAb sequencing.

**Primer name**	**Secuence**	**Use**
Oligo dT	**5´-d(TTTTTTTTTTTTTTTTTT)-3´**	RT-PCR (mRNA retrotranscription to cDNA)
VH1	**5′-CATGCCATGACTCGCGGCCCAGGCGGCCATGGCCCAGGTGCAGCTGGTGCAGTCTGG-3**	VHH PCR (Forward primer in amplification of VH1 SdAb family)
VH3	**5′-CATGCCATGACTCGCGGCCCAGGCGGCCATGGCCGAGGTGCAGCTGGTGGAGTCTGG-3′**	VHH PCR (Forward primer in amplification of VH3 SdAb family)
VH4	**5′-CATGCCATGACTCGCGGCCCAGGCGGCCATGGCCCAGGTGCAGCTGCAGGAGTCGGG-3′**	VHH PCR (Forward primer in amplification of VH4 SdAb family)
JH	**5′-CCACGATTCTGGCCGGCCTGGCCTGAGGAGACRGTGACCTGGGTCC-3′)**	VHH PCR (Reverse primer in amplification of all SdAb families)
Ompseq	**5′-AAGACAGCTATCGCGATTGCAG-3′**	Sequencing of SdAbs clones in pComb3XSS or pET-Mod vectors

Table S2: Flow cytometry antibodies used in CAR-T cell phenotyping and other *in vitro* assays.

Antigen	Clone	Fluorochrome	Reference, RRID*	Isotype	DF*	Panel
EGFR	AY13	BV421	(BioLegend Cat# 352911, RRID:AB_2562213)	IgG1	200	SUB*/ACT*/EXH*
CD8	RPA-T8	BV510	(BioLegend Cat# 301047, RRID:AB_2561378)	IgG1	100	SUB/ACT/EXH
CD4	OKT4	FITC	(BioLegend Cat# 317408, RRID:AB_571951)	IgG2b	150	SUB
CD3	OKT3	PE/Cy7	(BioLegend Cat# 317333, RRID:AB_2561451)	IgG2a	2000	SUB
CCR7	G043H7	PE	(BioLegend Cat# 353204, RRID:AB_10913813)	IgG2a	62,5	SUB
CXCR3	G025H7	APC	(BioLegend Cat# 353708, RRID:AB_10983064)	IgG1	50	SUB
CD45RA	HI100	PerCP/Cy5.5	(BioLegend Cat# 304121, RRID:AB_893358)	IgG2b	100	SUB
CD3	UCHT1	FITC	(BioLegend Cat# 300406, RRID:AB_314060)	IgG1	150	ACT/EXH
CD69	FN50	PE	(BioLegend Cat# 310906, RRID:AB_314841)	IgG1	50	ACT
HLA-DR	L243	PE/Cy7	(BioLegend Cat# 307615, RRID:AB_493589)	IgG2a	1000	ACT
ICOS	C398.4A	PerCP/Cy5.5	(BioLegend Cat# 313518, RRID:AB_10641280)	Hamster IgG	50	ACT
CD137	4B4-1	APC	(BioLegend Cat# 309809, RRID:AB_830671)	IgG1	300	ACT
LAG3	T47-530	PE	(BD Biosciences Cat# 565617, RRID:AB_2889327)	IgG1	250	EXH
PD1	EH12.2H7	PerCP/Cy5.5	(BioLegend Cat# 329913, RRID:AB_1595561)	IgG1	200	EXH
TIGIT	A15153G	PE/Cy7	(BioLegend Cat# 372713, RRID:AB_2632928)	IgG2a	50	EXH
TIM3	F38-2E2	APC/Cy7	(BioLegend Cat# 345025, RRID:AB_2565716)	IgG1	50	EXH
**Antigen**	**Clone**	**Fluorochrome**	**Brand**	**Isotype**	**DF**	**Use**
CD33	WM53	BV510	BD Bioscience	IgG1κ	100	AML cell staining
HA Tag	16B12	PE	Biolegend	IgG1κ	100	SdAb binding in AML cells
EGFR	AY13	APC	Biolegend	IgG1	200	CAR expression/ Lentivirus titration

* Abbreviations: RRID: Research Resource Identifiers; DF: Dilution factor in the staining mix (solvent PBS, BSA1%, EDTA 2mM, beriglobin 1/100); SUB: Subpopulations panel; ACT: Activation panel; EXH: Exhaustion panel.

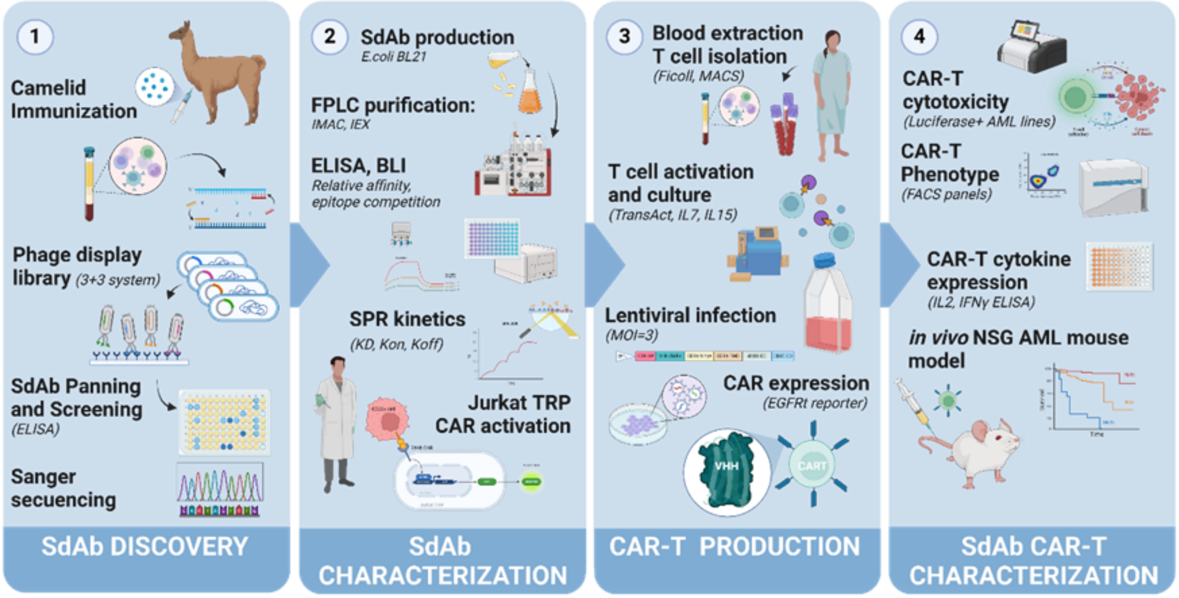

Figure S1: Schematic representation of the main processes for SdAb-based CAR-T cell generation. **(1) Immunization and SdAb library generation**: Llamas were immunized with a series of antigens, including CD33. The immune response was evaluated using indirect ELISA to determine the optimal time for peripheral blood extraction. Total RNA was extracted from isolated peripheral blood mononuclear cells (PBMCs) to generate SdAb DNA libraries through reverse transcription-polymerase chain reaction (RT-PCR). SdAb-specific PCR products were cloned into the pCOMB3XSS phagemid. E. coli TG1and ER2738 strains were transformed, followed by infection with the M13KO7 helper phage. Multiple panning rounds were conducted, and ELISA screening identified specific binders for both antigens. Single positive clones were sequenced and subsequently subcloned into a prokaryotic expression vector for protein production in E. coli BL21. Purification was achieved using fast protein liquid chromatography (FPLC) with immobilized metal affinity chromatography (IMAC) and ion-exchange chromatography (IEC). **(2) SdAb characterization:** Purified SdAbs underwent thorough characterization, including the assessment of affinity, kinetic constants, and epitope competition through ELISA, surface plasmon resonance (SPR) using Biacore™ X100, and bio-layer interferometry (BLI) using Octet® N1. 5 different candidates were used as recognition moieties in CAR constructs and CAR activation profile was evaluated in a reporter T cell line (Jurkat TRP). **(3) CAR-T cell production:** T cells were isolated using the Ficoll method and magnetic cell sorting with AutoMACS® NEO to separate CD4+ and CD8+ PBMCs. They were then expanded using TransAct™ (CD3/CD28 stimulating beads) in the presence of IL7 and IL15, and subsequently infected with lentiviral vectors carrying CAR constructs. **(4) CAR-T cell characterization:** Characterization of CAR-T cells included phenotyping using flow cytometry, measuring IL2, IFNγ and TNFα production through ELISA, and assessing cytotoxicity against AML cell lines using the GloMax® bioluminescence assay. The immunodeficient NSG AML (MOLM13) mouse model was employed to determine the efficacy of SdAb-based CAR-T cells in comparison to ScFv-based CAR-T cells.

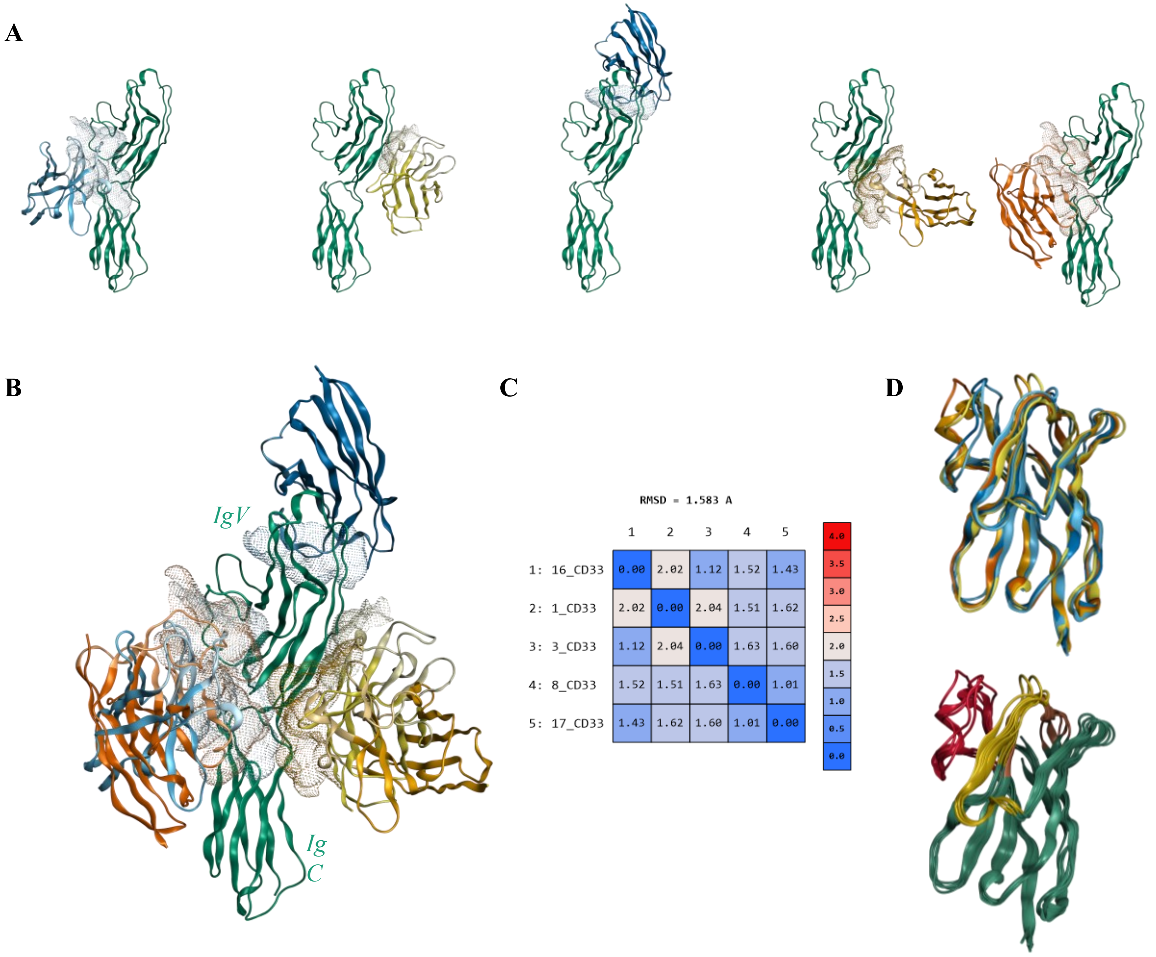

Figure S2: *In silico* prediction of CD33-SdAb interactions through structural modeling and molecular docking. **(A-B)** Predicted binding poses for Nb1 (light blue), Nb3 (yellow), Nb8 (dark blue), Nb16 (light orange), and Nb17 (dark orange) with the three-dimensional structure of CD33 (green), as represented by PDB entry 5IHB (amino acids 21 to 232), both separately (**A**) and in combination (**B**). The SdAb structures were modeled using the SAbPred NanoBodyBuilder2 tool, and molecular docking was performed with the Molecular Operating Environment (MOE) protein-protein docking tool, restricting the docking site to the SdAb CDRs. The dotted surfaces represent predicted interactions between the SdAb CDRs and CD33. The resulting scores (S) and RMSD values were as follows: -73.08/0.81 (Nb1), -64.75/0.43 (Nb3), -56.34/1.48 (Nb8), -60.43/0.49 (Nb16), and -63.25/1.99 (Nb17). **(C)** Similarity plot based on RMSD values between the SdAbs. **(D)** Alignment and superposition of the SdAb structures. Top: colored by respective colors—Nb1 (light blue), Nb3 (yellow), Nb8 (dark blue), Nb16 (light orange), and Nb17 (dark orange). Bottom: colored by regions—CDR3 (red), CDR2 (yellow), CDR1 (orange), and framework regions (FR) (green).

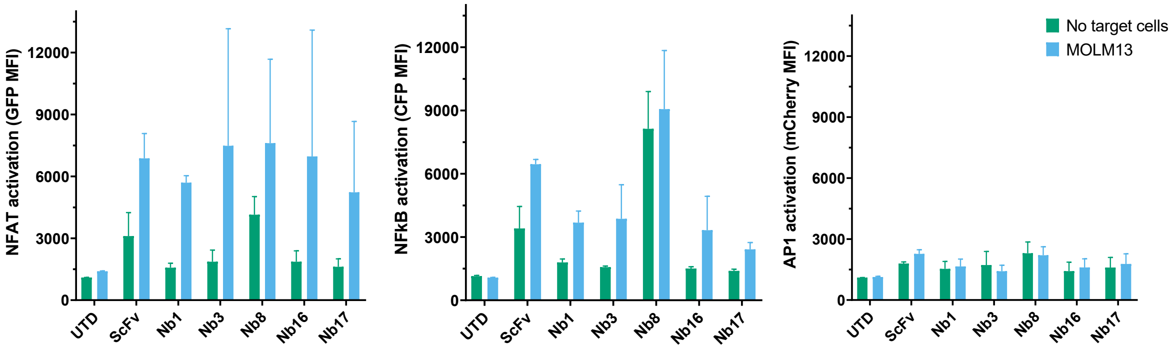

Figure S3: Jurkat TRP activation assays: MFI of each activation channel, from left to right: NFAT (GFP reported), NFkB (BFP reported) and AP1 (mCherry reported) mediated activation of untreated cells (green bars) and co-cultured with MOLM13 in a 1:1 ratio (light blue bars).

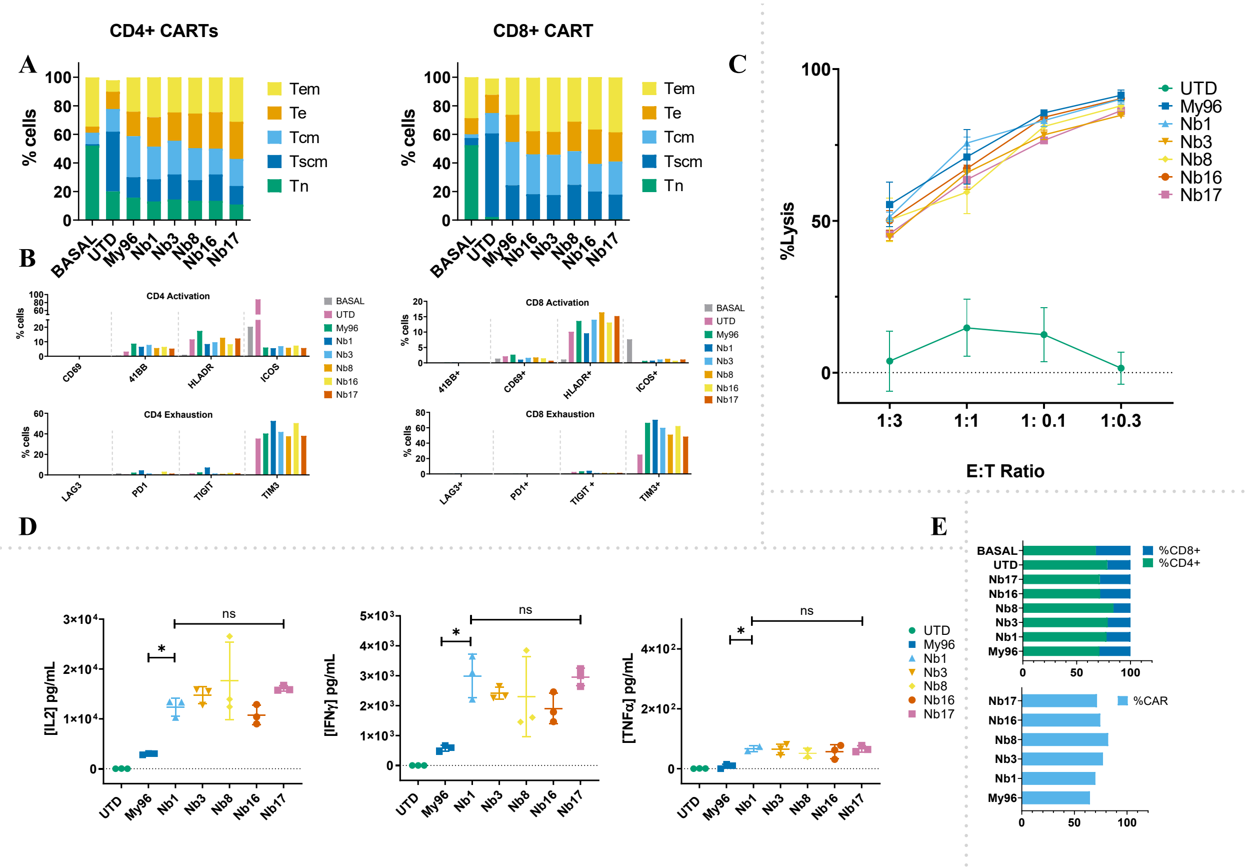

Figure S4: Phenotypical and functional characterization of the 5 SdAb-based CAR-T cells (including Nb1, Nb3, Nb8, Nb16 and Nb17) produced from a healthy donor. **(A)** T cell subpopulation phenotypes for CD4+ and CD8+ CAR T cells. **(B)** Exhaustion and activation markers expressed on CD4+ and CD8+ CAR T cells. **(C)** CAR-T cell cytotoxicity in MOLM13 cell line. **(D)** CAR-T cell cytokine production, from left to right: IL2, IFNγ, TNFα. **(E)** Distribution of CD8+ and CD4+ CAR-T cells and percentage of CAR+ cells in for each CAR construct.

A

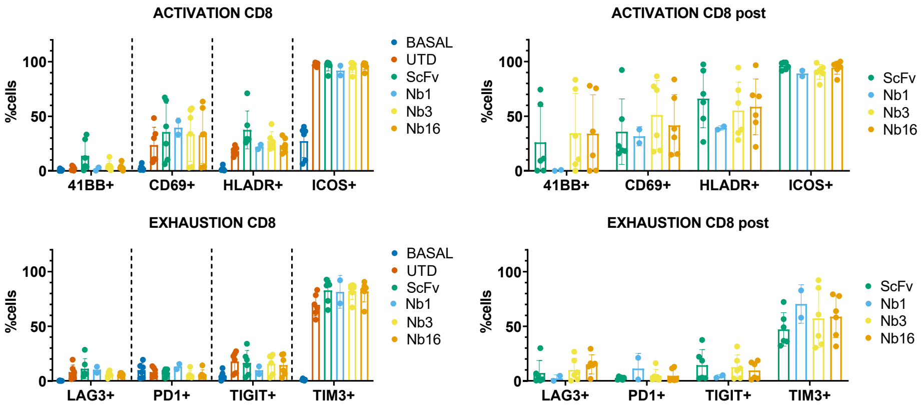

B

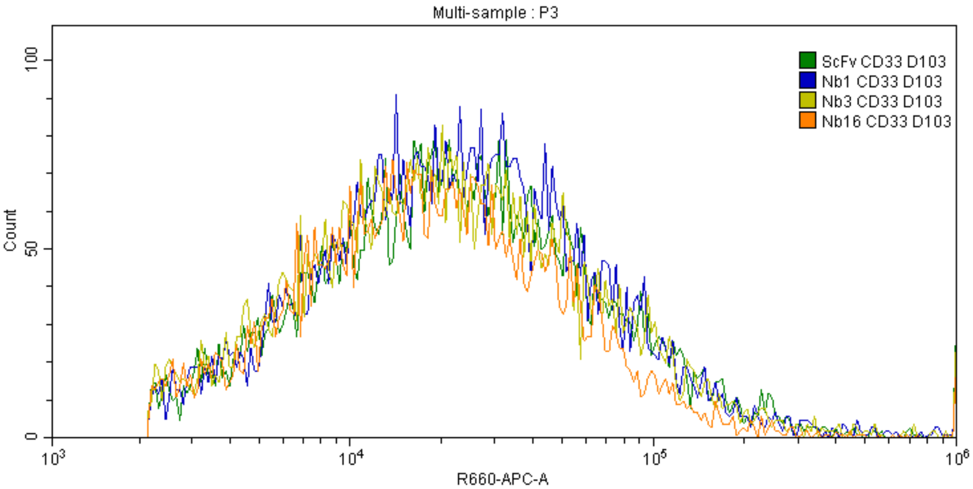

Figure S5: SdAb-CAR-T cell characterization assays. **(A)** Phenotypical characterization of the activation and exhaustion markers of CD8+ CAR T cells, at the end of the production (left, N=10) and after repeated stimulation (right "post" N=4). **(B)** APC (EGFRt staining) signal histograms representing CAR densities of CAR-T cells, MFI is very similar in all cases.

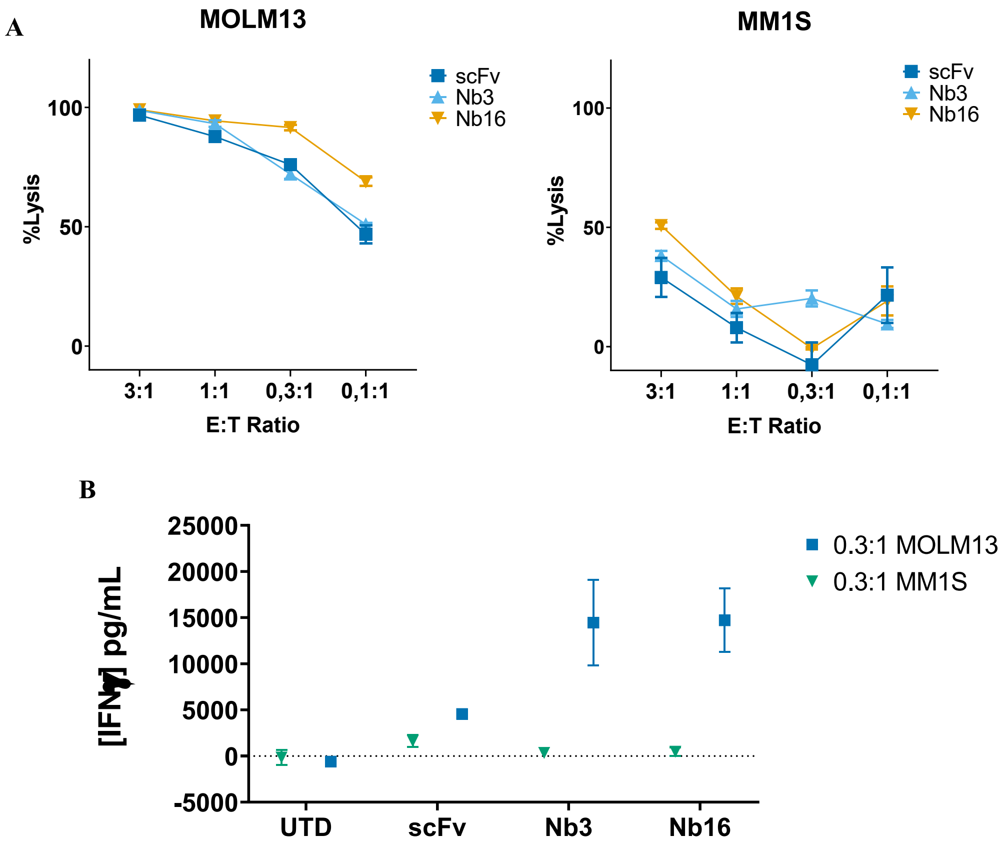

Figure S6: CAR-T cell activation over MM1S control cell line (CD33-). **(A)** Cytotoxicity of CAR-T cells on MOLM13 (CD33+) and MM1S (CD33-) cells. **(B)** IFN-γ secretion of CAR-T cells co-cultured with MOLM13 and MM1S cells.

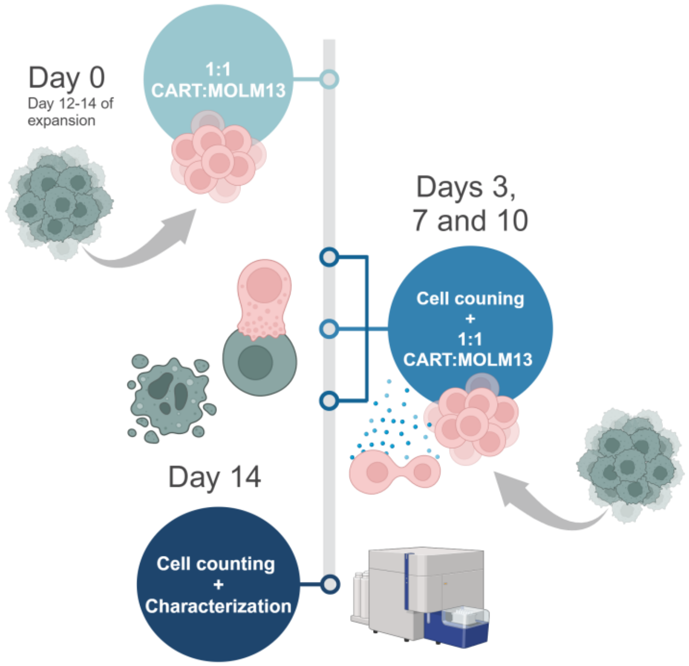

Figure S7: Schematical representation of the repeated stimulation of CAR-T cells using MOLM13 cell line.

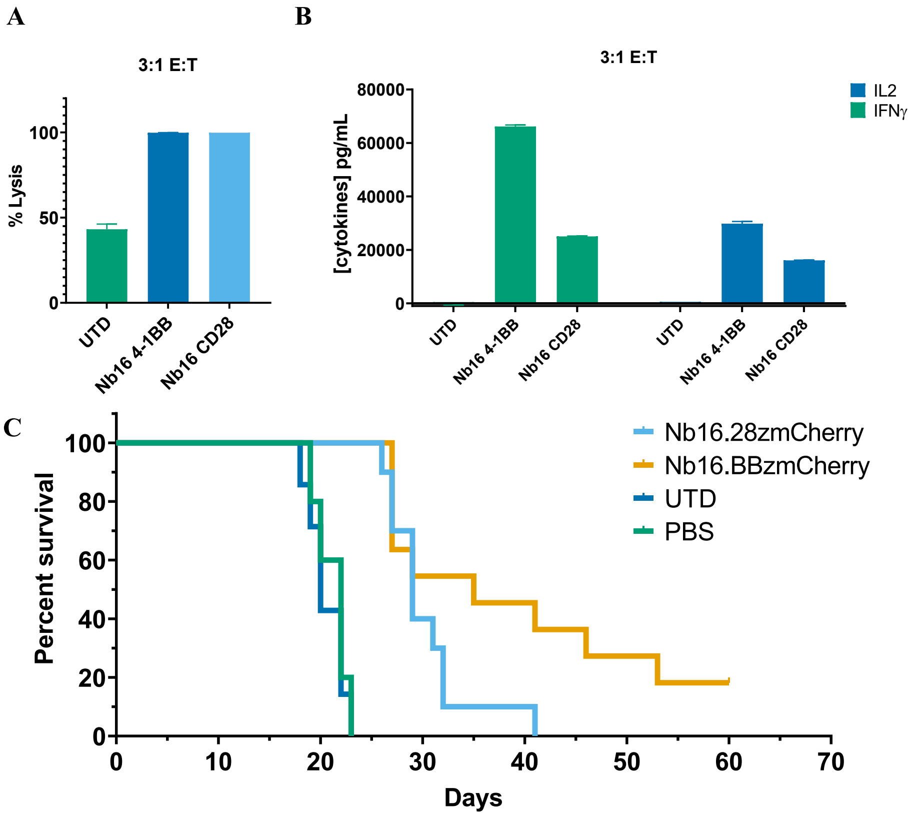

Figure S8: Evaluation of Nb16-CAR co-stimulation domains. (**A-B**) Cytotoxicity and cytokine production (IL2 and IFNγ) observed for CAR-T cells with CD28 or 4-1BB co-stimulation, co-cultured with MOLM13 cells in a 3:1 E.T ratio. **(C)** *In vivo* survival of AML (MOLM13) xenografted mouse model treated with Nb16-based CAR-T cells using 41BB or CD28 co-stimulatory domains.

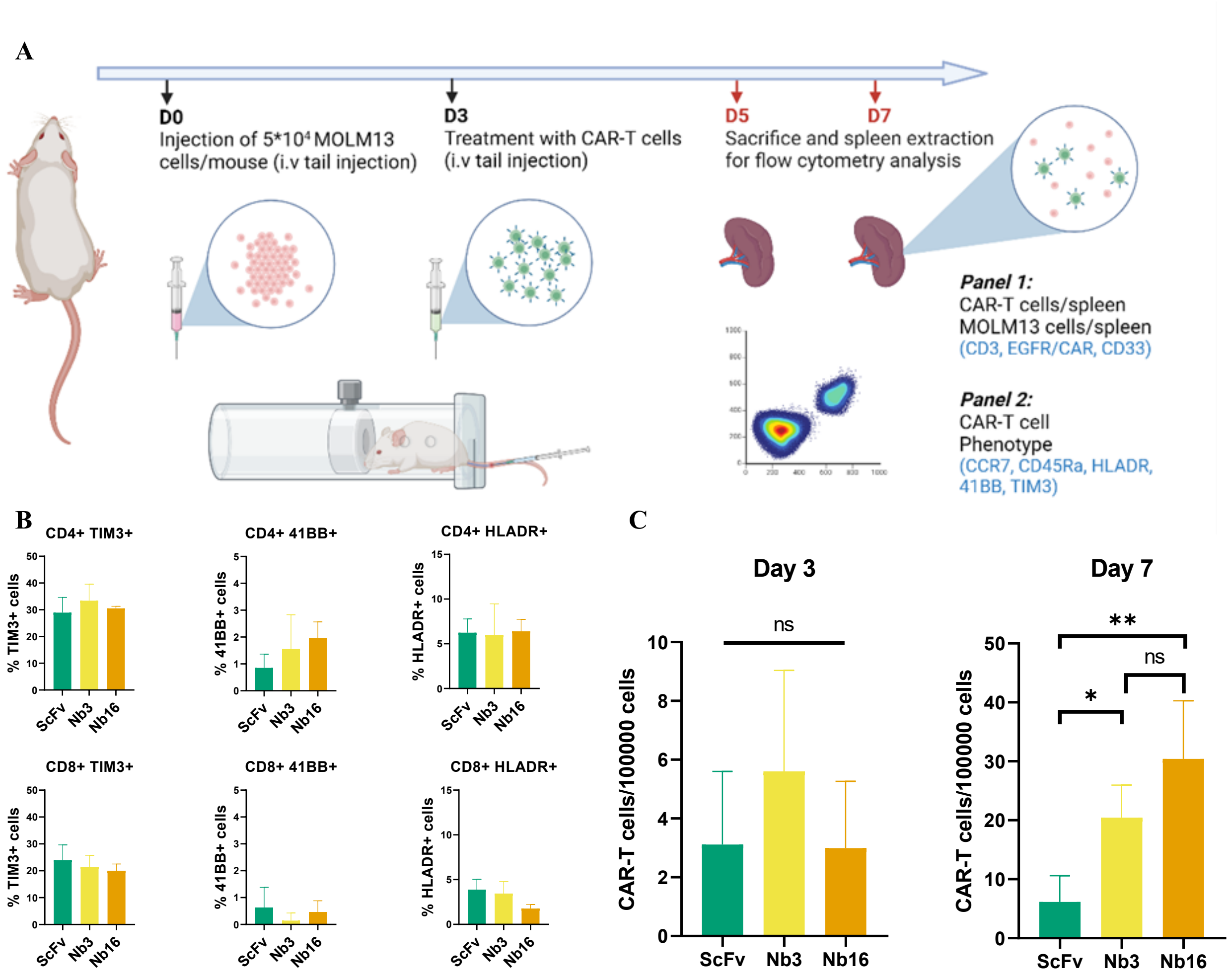

Figure S9: *In vivo* phenotype and persistence of CAR-T cells. (A) Timeline scheme for the MOLM13 xenograft *in vivo* experiment with sacrifice for CAR-T cell phenotyping. (B) Exhaustion and activation markers expressed on CAR-T cells isolated from the spleen of treated mice 3 days after CAR-T cell injection (N=4). No significant differences were detected in a one-way ANOVA test with multiple comparisons. (C) Number of CAR-T cells isolated per 1000000 total cells from the spleen of CAR-T cell treated mice (N=4). left: sacrifice 3 days after injection, right: sacrifice 7 days after injection. Data passed Shapiro-Wilk normality test (alpha=0,05) and one-way ANOVA with multiple comparisons was used to compare groups. **: p>0,01; *:p>0,05; ns: non-significant.

A

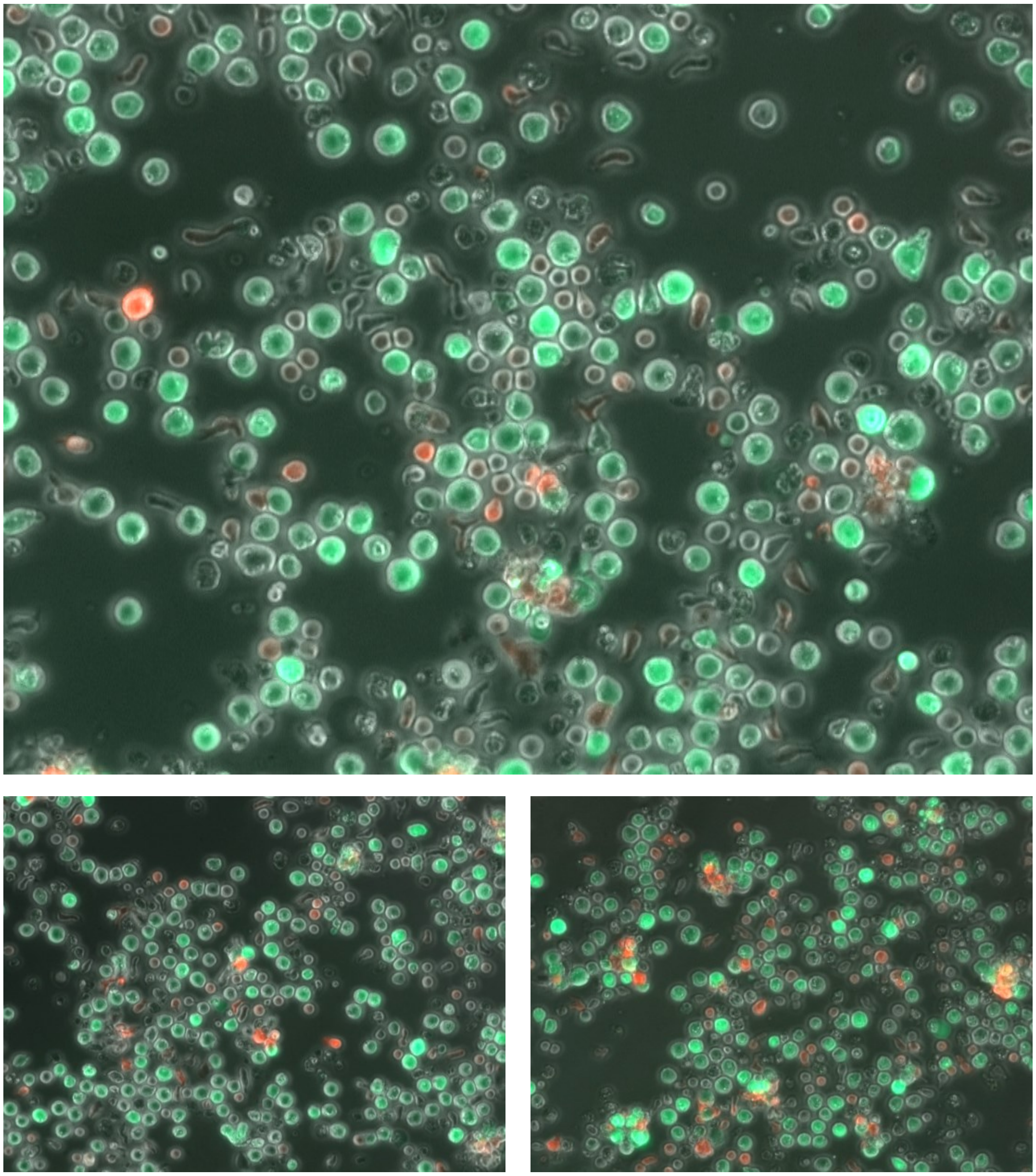

B

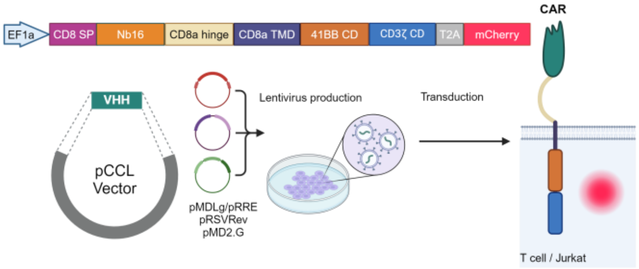

Figure S10: Visualization of Nb16-CAR-T cells co-cultured with MOLM13 cells. **(A)** Fluorescence microscopy timelapses of the Nb16-CAR-T cells (RED: mcherry+) co-cultured with MOLM13 (GREEN: GFP+) in a 0,3:1 E:T ratio after 4 hours of incubation. **(B)** Representation of the mCherry reported Nb16 CAR construct.
